# ISGylation and E3 ubiquitin ligases: an Atlantic salmon genetic perspective

**DOI:** 10.3389/fimmu.2025.1554680

**Published:** 2025-06-24

**Authors:** Unni Grimholt, Hilde Sindre, Arvind Y. M. Sundaram

**Affiliations:** ^1^ Fish Health Research Section, Norwegian Veterinary Institute, Ås, Norway; ^2^ Department of Medical Genetics, Oslo University Hospital, Oslo, Norway

**Keywords:** Atlantic salmon, ISAV, IFN gamma, *ISG15*, ISGylation, E3 ubiquitin ligases, HERC, RBR family

## Abstract

**Introduction:**

In mammals, the ubiquitin–proteasomal pathway plays a key role in the host antiviral response by targeting viral genes for degradation. Here, E1-activating enzymes, E2-conjugating enzymes, and E3 ligases attach the ubiquitin chain to molecules to be functionally modified or destined for degradation. One specialized version of this pathway is performed by ISG15, a ubiquitin-like protein modifier that works through a process called ISGylation. In mammals, ISGylation involves specialized E1–E3 molecules and specialized mammalian ISG15 “deubiquitinases” also exist. Targeting host and viral proteins, ISG15 can inhibit the release of viral particles or hinder viral replication, thereby exerting strong antiviral effects. In Atlantic salmon, endothelial cells from the heart is a main target for infectious salmon anemia virus (ISAV).

**Results:**

Here, we established a new cell line from Atlantic salmon heart tissue denoted ASH2-2, which has endothelial-like characteristics and is permissive for infection with ISAV and other salmonid viruses. We used this cell line as a model to compare the effect of recombinant interferon gamma (rIFNg) and ISAV on genes potentially involved in the ubiquitin–proteasome pathway. ASH2–2 cells have a response profile matching endothelial cells and respond quickly to ISAV infection with upregulation of viral sensors such as DHX58, MDA5, and MX transcripts. Two *ISG15* genes are strongly upregulated 48 h post-infection (p.i.) while other ubiquitin genes were unaffected. Related to the mammalian E3 ligases known to be ISGylated, phylogenetic analysis identified two additional teleost-specific HERC8 and HERC9 clusters in addition to the clade previously defined as HERC7. Duplicate genes for Atlantic salmon *HERC7* and *HERC9* are both more upregulated by virus and rIFNg at 24 h p.i. as opposed to *HERC8* genes. Early regulation of the ISGylation process is also indicated by a strong upregulation of one *USP18* duplicate already at 4 h p.i.

**Conclusion:**

In conclusion, we expand the list of teleost genes potentially involved in the ubiquitin–proteasome pathway in cells from a main target organ. Our results highlight the need for functional studies to clarify the roles of these candidates.

## 1 Introduction

Protection against viral infection relies on the host being able to sense infection and induce a protective immune response. In turn, pathogens have evolved multiple strategies to counteract detection by their hosts promoting the evolution of host immune genes. One such example is the ubiquitin(-like) proteasome system, which regulates the innate sensing pathways initiated by pattern recognition receptors (PRRs), ultimately coordinating an effective antiviral immune response.

PRRs detect conserved viral pathogen-associated molecular patterns or so-called PAMPs (1). Zinc finger NFX1-type containing 1 (*ZNFX1*), stimulator of interferon response cGAMP interactor 1 (*STING1*), retinoic acid-inducible gene I (*RIG-I*, alias *DDX58*), and melanoma differentiation-associated gene 5 (*MDA5*, alias *IFIH1*) all express PAMPs that interact with mitochondrial antiviral signaling protein (*MAVS*), with subsequent induction of interferon-stimulated genes (ISGs) (2–4).

Posttranslational modification of RIG-1 and downstream signaling proteins by different types of ubiquitination regulates their activity. The PAMP *LGP2* (Laboratory of Genetics and Physiology 2) does not bind to MAVS but regulates the activities of RIG-I likely through various mechanisms including the prevention of RIG-I binding to MAVS. In peripheral tissues, sensing of viral infection results in expression of interferons and pro-inflammatory cytokines that attract immune cells to the infected region. They also induce expression of a wide array of ISGs that inhibit viral replication and spread (5). One such ISG is the myxovirus-resistance (*Mx*) gene, an IFN-inducible GTPase. As opposed to humans, Atlantic salmon has nine *Mx* genes where some respond more to type I IFN while others respond more to type II IFN (6).

Once the virus has entered the cell and starts replicating, the host tries to eliminate viral proteins through the ubiquitin–proteasome pathway [reviewed in (7)]. Ubiquitination is the first step in this protein degradation process. In humans, ubiquitin is encoded by the four genes *UBB*, *UBC*, *UBA52*, and *RPS27A* where *UBB* and *UBC* encode multimeric ubiquitin chains while *UBA52* and *RPS27A* are associated with the ribosomal proteins L40 and S27A, respectively (8). Other ubiquitin-like genes encoding proteins with one or several ubiquitin-like (UbL) domain are *NEDD8*, *SUMO1*, and *ISG15* (9, 10).

A classical view has been that Ub tagging is required for targeting proteins for 26S proteasomal degradation. Recently, this view has been challenged where multiple authors have shown that alternative Ub-independent proteasome degradation also occurs (11, 12). Additionally, Ub-like domains are shown to bind to 26S proteasomes and activate proteasomal degradation (13, 14). We therefore use Ub/L hereafter to underline that either Ub or Ub-like conjugation can occur.

Initially, a single Ub/L chain is attached to an E1 enzyme, which then transfers this Ub chain to an E2 enzyme and subsequently to an E3 ligation enzyme. The E3 ligation enzyme provides the specificity for further addition of Ub to target proteins prior to proteasomal degradation. In mammals, the ubiquitin-like molecule interferon-stimulated gene 15 (*ISG15*) is attached to various targets in a process called ISGylation (15, 16). ISG15 attachment involves the ubiquitin-activating enzyme E1 (UBE1L), the ubiquitin-binding enzyme E2 (UBCH8), and some E3 ubiquitin ligases such as the ring finger protein (RNF) 213, ARIH1, TRIM25, and HERC5 (16–18). ISGylation not only modifies key molecules in immune signaling pathways but also inhibits viral replication through ISGylation of viral proteins. ISG15 can also exist in a free unconjugated format with various biological functions such as regulating interferon expression or signaling and mediating degradation of RIG-1. In Atlantic salmon, the *ISG15* gene responds similarly to both interferon and infection by ISAV (19–21). Atlantic salmon ISG15 has also been shown to bind viral ISAV proteins such as, for instance, segment 8 open reading frame protein 2 (22).

The E3 ubiquitin ligase enzymes provide target specificity and 919 different human E3 enzymes. The various E3 ligases have traditionally been classified into three main families, i.e., RING (really interesting new genes), HECT (homologous to the E6-AP carboxyl terminus), and RBR (ring-between-ring) domain-containing E3 ligases [reviewed in (23)]. RING ligases facilitate the direct transfer of Ub/L chains from the E2 cysteine to the substrate while HECT and RBR ligases transfer the Ub/L chain from the E2 enzyme first to the E3 ligase prior to transferring it to the substrate.

Tripartite motif (*TRIM*) genes belong to the E3 ligase RING family. Humans harbor 80 *TRIM* genes further subdivided into nine subgroups depending on their C-terminal domains, subcellular location, and functionality (24). Several human *TRIM* genes are shown to regulate host antiviral activities against, for instance, influenza virus (25, 26). For instance, *TRIM25* has been shown to mediate ubiquitination of RIG-1, leading to type 1 interferon production (27). As a countermeasure, the NS protein of the influenza virus targets TRIM25 to evade RIG-1 recognition (28). In addition to *TRIM25* and *TRIM39*, teleosts have a unique subgroup denoted finTRIMs where many are upregulated upon infection in both zebrafish and rainbow trout (29–32).

Some ring finger protein genes also belong to this RING family of E3 ubiquitin ligases. *RNF213* is an interferon upregulated gene that acts as an ISG15 interactor and cellular sensor of ISGylated proteins with broad antimicrobial activity in humans (18, 33). *RNF213* is also upregulated by interferon in teleosts (34, 35). Another member of this family (*RNF39*) activates the ubiquitin–proteasome pathway and thus inhibits RIG-I-like receptor-dependent antiviral immunity (36).

HECT E3 ligase family members are characterized by the presence of a HECT domain and one or more RCC1-like domains. In addition to ubiquitylating proteins for degradation by the 26S proteasome, HECT E3 enzymes regulate the trafficking of many receptors, channels, transporters, and viral proteins [reviewed in (37)]. *HERC* genes belong to this family with humans having two large genes containing multiple RCC1 domains (*HERC1* and *HERC2*) and four small genes containing single RCC1 domains (*HERC3-6*) (38). Both *HERC5* and *HERC6* are upregulated by interferon, where the major ISG15 E3 ligase is human *HERC5* and *HERC6* in mice (39–41). In humans, *HERC5* is shown to attenuate influenza A by catalyzing ISGylation of the viral NS1 protein (42). A teleost-specific HERC gene denoted *HERC7* was recently described in carp and zebrafish with four genes in zebrafish (43, 44). Carp *HERC7* has an E3 ligase activity for conjugation of both ubiquitin and ISG15 while zebrafish *HERC7c* only displays the potential to transfer ubiquitin. Zebrafish *HERC7c* acts as a negative regulator of fish interferon responses by targeting STING, MAVS, and IRF7 for protein degradation.

RBR domain-containing E3 ligases, the third family of E3 ligases, contain three zinc-binding domains termed RING1, in between RING (IBR) and RING2 domains (collectively called the RBR module), and are described as RING/HECT hybrid E3 ligases (45). Humans harbor 14 RBR members including four Ariadne ligases, Parkin, and six RNF ligases [reviewed in (46)]. Several of these RBR ligases affect immunity against viruses. For instance, ARIH1 inhibits influenza A virus replication and facilitates RIG-1-dependent immune signaling (47). RNF144 promotes antiviral responses by modulating STING ubiquitination (33).

Once conjugated to Ub/L chains, some proteins are targeted for degradation by the proteasome. The 26S proteasome consists of a 20S core proteasome and a 19S regulator. In mammals, the 19S regulator contains multiple ubiquitin binding sites, which regulate the proteasome conformation that activates unfolding, deubiquitination, and degradation of various protein substrates [reviewed in (48)]. Three of the 19S subunits function as Ub/L receptors. The subunit Rpn10 functions as the primary ubiquitin chain receptor and the two Rpn13 and Rpn1 subunits can cooperate with Rpn10 to enhance degradation of some proteins (49). Once deubiquitinated and unfolded, the protein is transferred to the 20S core for further degradation.

The 20S core particle has duplicate rings of seven alpha (PSMA) and beta (PSMB) subunits (50). Upon IFN stimulation, the PSMB5–7 subunits are exchanged with the major histocompatibility complex (MHC)-adapted subunits PSMB8–10 in humans. These IFN-induced subunits produce peptides that are optimally suited for binding to the antigen transporter TAP and to classical MHC class I (MHCI) molecules (51). In addition to the PSMB8 and PSMB9 subunits, teleosts have an additional unique PSMB9 subunit denoted PSMB12 as well as an additional PSMB10 subunit denoted PSMB13 (52). In Atlantic salmon, these PSMB8–10-like genes reside in duplicate MHCI regions on chromosome (chr.) 14 and chr.27 (53).

ISGylation is a reversible process where deubiquitinases or DUBs contribute in the deubiquitination process. In mammals, the ubiquitin-specific protease 18 (USP18) is specific for ISG15-conjugated proteins (54–56). In addition to the enzymatic activity, USP18 also interacts with the type I interferon receptor and shuts off downstream signaling.

MHC-compatible protein fragments resulting from proteasomal degradation are then selectively transported into the endoplasmic reticulum through the TAP1/TAP2 channel where they are loaded onto the awaiting MHCI molecule prior to transport to the cell surface for recognition by other immune cells [reviewed in (57)]. MHCI peptide loading and editing are assisted by various chaperones including tapasin-related (TAPBPR) and the aminopeptidase ERAAP molecules (58–61). Additional molecules, such as the tapasin-like (TAPBPL) molecules identified in teleosts and some tetrapods, may have as yet undefined roles in the peptide editing process (53). In Atlantic salmon, *TAP2* and *TAPBP* genes reside within the duplicate MHCI regions on chr.14 containing non-classical MHCI genes and chr.27 harboring the single classical MHCI gene denoted *UBA* (62). With the recent advances in understanding of the Ub/L-proteasome pathway, it is timely to reinvestigate these responses also in teleosts.

Here, we investigate these responses in a new Atlantic salmon cell model with endothelial-like characteristics originating from the heart, following infection with ISAV, an orthomyxovirus virus with eight negative stranded RNA segments (63). This allows us to assess the interactions between ISAV and host genes in the first model system from a target cell and target organ (64, 65). We compare the effect of viral infection against stimulation with recombinant interferon gamma (rIFNg) on gene expression unaffected by potential viral evasion strategies.

## 2 Material and methods

### 2.1 Animal husbandry and ethical considerations

Tissue for the development of primary cells was obtained from a healthy 15-g pre-smolt Atlantic salmon (*Salmo salar*) at a Norwegian salmon hatchery. A trained fish veterinarian euthanized the fish with an overdose of MS222 (Sigma-Aldrich, St. Louis, MO, USA) of 250 mg/L as recommended in the guidelines, before the whole heart was removed and incubated in medium consisting of Leibovitz-15 (L-15, Lonza, Basel, Switzerland) with the addition of 100 units of potassium penicillin and 100 μg of streptomycin sulfate (1% pen/strep, Lonza) and 20% fetal bovine serum (FBS, Lonza) and processed as described in Section 2.2 on the same day the tissue was harvested.

### 2.2 ASH2–2 cells and study design

The ASH2–2 cells were established by mincing the harvested heart into small pieces with a scalpel. Then, the cells were digested in 0.25% trypsin/EDTA (Lonza) in a sterile container under stirring for 1 h. The solution was then filtered through a cell strainer and transferred to a 15-mL Falcon vial, L-15 Leibovitz medium (#L1518, Merck AS, New Jersey, USA) was added, and the vial was centrifuged at 1,500 rpm for 10 min, to pellet dissolved cells. The cells were washed once with L15 and a new centrifugation was performed. Then, the cells were resuspended in L15 medium with the addition of 100 units of potassium penicillin, 100 μg of streptomycin sulfate (1% pen/strep, Lonza), and 20% FBS (Lonza), and were seeded into Falcon Primaria 6-well plates (Becton Dickson & Company) and incubated at 20°C without CO_2_ for several weeks until a cell monolayer was established. The medium was changed several times during the incubation period to remove dead and non-adherent cells floating in the medium. The cells were then subcultured as follows: The cell layer was washed with PBS, incubated with a small amount of trypsin/EDTA at room temperature until detachment. L-15 medium with 20% FBS was added and the cells were split 1:1 into new wells. In the next subculture, the cells were passaged 1:2 into a 25-cm^2^ Falcon Primaria cell flask. After 7–10 passages, the remaining cells represented a relatively homogeneous monolayer of cells. At passage 10, the amount of FBS in the medium was reduced from 20% to 10% for further maintenance, and at this point, several vials for cryopreservation were also secured. Shortly, the cells from a 25-cm^2^ Falcon Primaria cell flask were treated with trypsin/EDTA, resuspended in L-15 with 2% FBS, and centrifuged at 1,500 rpm for 10 min at 4°C. The cell pellet was dissolved in ice-cold L-15 medium with 50% FBS and placed on ice. Then, a mixture of almost freezing 20% dimethyl sulfoxide (DMSO) in L15 with 50% FBS (stored at −20°C before addition) was carefully added by very gentle mixing. The vials were then stored first for 24 h in a Styrofoam box at −80°C before transferring them to a liquid nitrogen tank.

The cells were demonstrated to be permissive to ISAV through experimental infection. ASH2–2 cells were passaged in 96-well cell plates (Corning) with L15 and 10% FBS as described above and incubated at 20°C without CO_2_ for 2 days until approximately 80% confluence. A plate of the ISAV-permissive ASK cells (ATCC CRL-2747) was included as a positive reference. Then, standard inoculation of a 10-fold dilution series of ISAV Glesvær was performed as described in the WOAH manual (66). The well plates were then incubated for 7 days. Virus-infected cells were visualized by microscopy following indirect fluorescent antibody test (IFAT) using an anti-ISAV monoclonal antibody (#P10, Aquatic Diagnostics Ltd, UK). In the same setup, the cells were also shown to be permissive to viral hemorrhagic septicemia virus (VHSV) and infectious pancreatic necrosis virus (IPNV) (data not shown).

ISAV Glesvær passage 3 titer 10^6,3^/mL was propagated in ASK cells as described previously for the SHK-1 cells (67) and titered as described in the WOAH manual (66) with some modification. In short, a 10-fold dilution series was performed in 96-well plates using ASK cells. Visualization of positive staining was done as described before, and the titer was calculated with tissue culture infectious dose (TCID) 50 end point titration as describes by Kärber (68).

The rIFNg batch and amount used here have been used previously and shown to provide a proper induction of expected molecules (69). In brief, the mature Atlantic salmon IFNg sequence of NM_001171804.1 inserted into pET15/D-Topo was purchased from GeneArt (Thermo Fisher Scientific, MA, USA) and transfected into *Escherichia coli* BL21 DE3 (Thermo Fisher Scientific, #C600003). Recombinant protein was isolated and purified following an established protocol (70). The purity of rIFNg was checked on a 4%–12% precast sodium dodecyl sulfate–polyacrylamide gel electrophoresis (SDS-PAGE) gel (Thermo Fisher Scientific) stained with SimplyBlue (Thermo Fisher Scientific). Protein concentration was measured with a Qubit Protein Assay kit (#Q33211, Thermo Fisher Scientific).

ASH2–2 cells in passage 27 were seeded into 25 cm^2^ flasks using trypsin/EDTA totaling 9.4 × 10^5^ cells per 25 flask used in this RNA-Seq study. Cells were grown at 15°C overnight in L-15 Leibovitz medium with L-glutamine (#L1518, Merck AS) supplemented with 5% FBS and gentamicin.

For ISAV infection, cells grown overnight in L15 supplemented media were exchanged with serum-free media containing ISAV isolate Glesvær at 0.1 multiplicity of infection (MOI). Cells were incubated at 15°C for 4 h before they were washed in PBS and returned to serum-supplemented L15 media. Five ASH2–2 flasks infected with ISAV were harvested 4 h post-infection (p.i.) (ISAV-4h samples) and five flasks were harvested 48 h p.i. (ISAV-48h samples). Another five ASH2–2 flasks were stimulated with 1 ng/mL culture media of rIFNg and harvested after 48 h (IFNg-48h samples). Negative controls were five flasks harvested after 4 h (C-4h samples) and five flasks harvested after 48 h (C-48h samples).

### 2.3 RNA-Seq library preparation and sequencing

Cells from five ASH2–2 flasks from each group were washed twice with ice-cold PBS, trypsinized using 1 mL of 0.25% EDTA-Trypsin and transferred to Eppendorf tubes. Cells were harvested at 2,500 × *g* for 5 min at 4°C, and resuspended in 350 μl of RLT buffer supplied in the RNeasy Mini kit (#74104, QIAGEN, Hilden, Germany). RNA was isolated including a DNase I treatment step according to the manufacturer’s protocol, (#79254, QIAGEN). RNA quantity and quality were analyzed using a 4150 Tapestation (Thermo Fisher Scientific) according to the manufacturer’s protocols. RNA quantity ranged from 174 to 582 ng/μL with RIN values of 7.4–10.0.

RNA-Seq libraries were prepared using a TruSeq stranded total RNA prep kit (Illumina, San Diego, CA, USA) targeting the poly-A tail to enrich mRNAs. A total of 25 barcoded libraries were pooled together and sequenced on a NovaSeq 6000 SP Flowcell (Illumina) employing 150-bp paired-end sequencing. Library preparation and sequencing were performed at the Norwegian Sequencing Center, Oslo, Norway.

### 2.4 Illumina data analyses

RNA-Seq analysis: From the raw sequenced data, adapters and low-quality reads were trimmed/removed using BBduk [BBMap v34.56 (71);] using “ktrim = r k = 23 mink = 11 hdist = 1 tbo tpe qtrim = r trimq = 15 maq = 15 minlen = 36 forcetrimright = 149” as parameters. Cleaned reads were aligned against the ENSEMBL Atlantic salmon genome (Salmo_salar.ICSASG_v2) using Hisat2 v2.1.0 (release 104 annotation - Salmo_salar.ICSASG_v2.104.gtf) [GCA_000233375.4 (72);]. Fragments (read pairs) aligning to the 136,077 transcripts were counted using featureCounts v1.4.6-p1 (parameters: “-s 2 -p”) (73). Differential expression analysis was carried out using variance stabilizing transformed data using all cleaned reads in the DESeq2 v1.22.1 (74) package in R v3.5.1. Significance cutoff was set at an adjusted *p*-value (*p*-value adjusted for false discovery rate) of 0.05. Sequence data have been submitted to the NCBI Sequence Read Archive (SRA) under the BioProject accession number PRJNA1197162.

ISAV expression: To calculate the expression of eight ISAV genes, cleaned sequence data were aligned to the above reference using HiSat2 v2.1.0. Total number of reads aligned to each of the 13 genes was counted using featureCounts v1.4.6-p1 using a custom SAF file as defined by the tool guide. In order to compare the samples, FPKM values were calculated from the raw count data using the standard FPKM formula.

### 2.5 Data mining

Genes of interest were further investigated using the Ensembl accession numbers provided in the RNA-Seq analysis. Gene duplications were identified through tblastn search using translated amino acid sequences against a new Atlantic salmon genome assembly (GCA_905237065.2) as the previous assembly used for RNA-Seq analysis was terminated.

Gene orthology was tested using clustal alignments (75) and phylogenetic analyses using MEGA 7 (76). In phylogenies, the percentage of trees in which the associated taxa clustered together are shown next to the branches. Initial trees for the heuristic search were obtained automatically by applying Neighbor-Join and BioNJ algorithms to a matrix of pairwise distances estimated using a JTT model, and then selecting the topology with a superior log likelihood value. The trees are drawn to scale, with branch lengths measured in the number of substitutions per site. All positions with less than 95% site coverage were eliminated. That is, fewer than 5% alignment gaps, missing data, and ambiguous bases were allowed at any position.

Gene homeologs were defined based on regional location (77). We use standardized nomenclature for gene duplicates originating from the fourth salmonid-specific 4R-WGD where one homeolog is given the extension –a and the other homeolog is given the extension –b. Some gene duplicates mostly originating from the 3R-WGD have been given the extension –like or –L, while gene copies of unknown location have consecutive numbers.

### 2.6 Expression in unstimulated tissues

Reads per kilobase per million mapped reads (RPKM) values were defined using CLC Genomic Workbench 6.0.5 [CLC Genomic workbench] and SRR transcriptome runs from a single individual (77) as follows: HK (head kidney) SRR1422860, gills SRR1422858, spleen SRR1422870, heart SRR1422862, gut SRR1422859, muscle SRR1422866, brain SRR1422856, liver SRR22865, nose SRR1422867, skin SRR1422869, ovary SRR1422871, and testis SRR1422872.1.

## 3 Results and discussion

**Figure 1:**
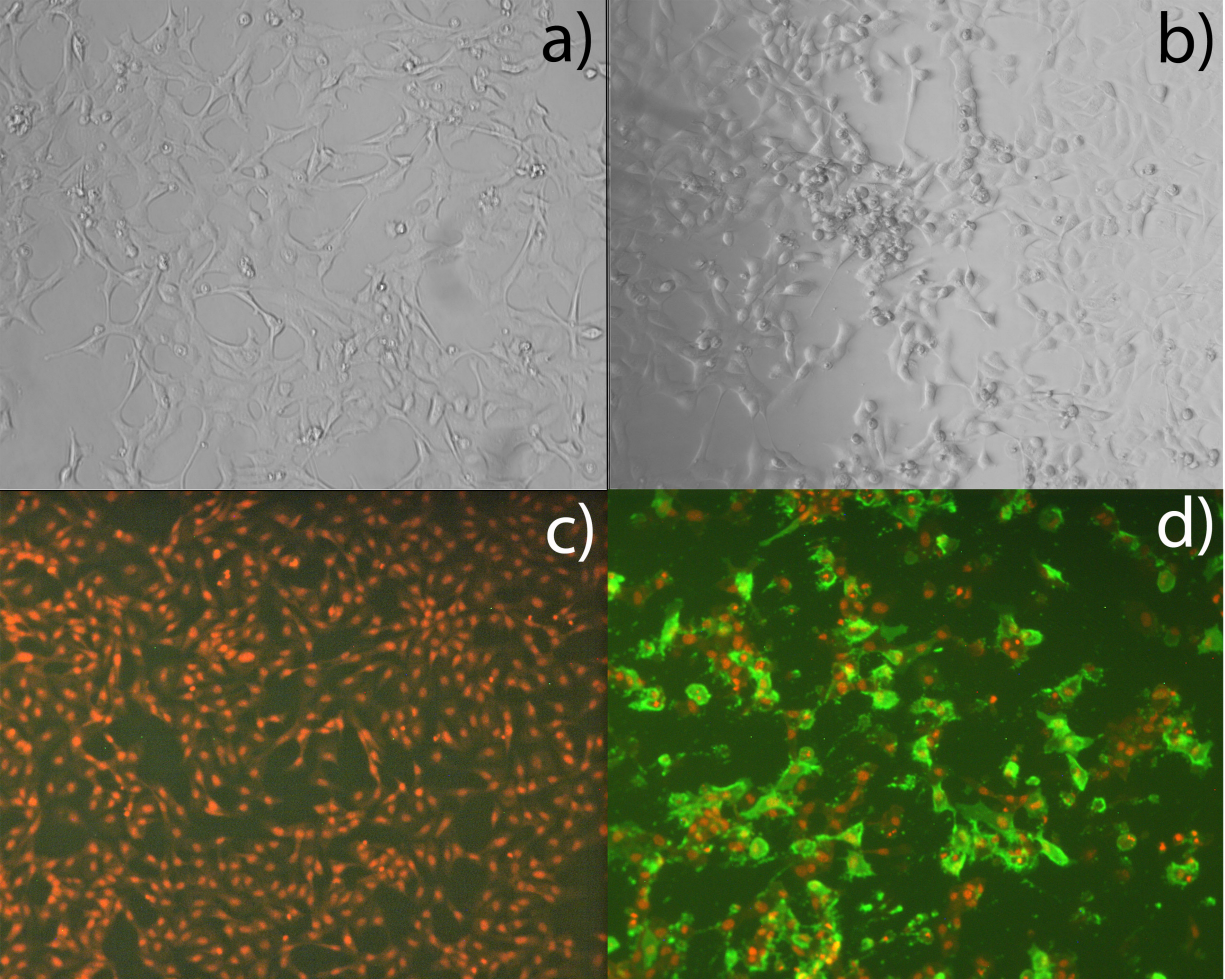
Susceptibility of ASH2–2 cells to ISAV. ASH2–2 passage 12, inoculated with mock control **(a, c)** and ISAV Glesvær **(b, d)** and incubated for 7 days. Viral protein visualized by IFAT (green); cell nucleus by propidium iodide (PI, red).

Our model system, i.e., the ASH2–2 cells, was made from heart tissue and was shown to be permissive to ISAV (**Figure 1**). A search for cell-specific markers showed that the ASH2–2 cell line most likely represents cardiac endothelial cells as defined by Andresen et al. (78) [**Supplementary File 1** (**SF1.1**)]. Typically, endothelial expressed genes are cadherin 5, vascular endothelial growth factor receptor, endothelial differentiation-related factor 1, and endothelial lipase.

To ensure that the observed effect was due to infection with virus, we also investigated expression of ISAV genes 4 h and 48 h p.i. Very few ISAV transcripts were present at 4 h p.i., but increased substantially at 48 h (**Table 1**; **Supplementary File SF1.2**).

**Table 1:** ISAV expression values (FPKM) in five replicate flasks at 4- and 48-h time points.

Gene	ISAV-4h replicates					ISAV-48h replicates				
	A1	A2	A3	A4	A5	B1	B2	B3	B4	B5
S1_PB2	4,117	4,095	4,295	4,347	2,795	70,081	74,798	68,878	63,427	79,673
S2_PB1	3,909	2,822	3,568	3,286	3,101	50,278	528,867	49,151	47,316	57,396
S3_NP	1,624	1,302	1,482	1,707	1,288	101,162	91,084	105,823	157,796	141,256
S4_PA	116	0	0	195	0	1,364	1,614	1,472	766	1,612
S5_F	0	0	0	0	0	118	172	102	88	40
S6_HE	11,945	8,571	9,509	8,456	7,780	398,093	400,083	393,485	325,192	307,914
S7_Orf1	430	230	0	0	0	1,127	1,428	1,291	1,167	391
S8_M	0	0	0	0	0	326	462	297	187	106

Expression values of eight ISAV genes representing each of the eight viral genome segments (110) in each of the five replicate flasks included in the ISAV-4h (A1–5) and ISAV-48h (B1–5) samples. Only open reading frame sequences were used to calculate FPKM (Fragments Per Kilobase of transcript per Million mapped reads) values. S1 is segment 1 (S1) of the polymerase PB2, S2 is the polymerase PB1, S3 is the nucleoprotein NP, S4 is the polymerase acidic protein PA, S5 is the fusion protein F, S6 is the hemagglutinin-esterase HE, S7 encodes the non-structural Orf1 protein NS1, while S8 encodes the matrix protein M. Accession numbers and length in base pairs used for each gene are shown in **Supplementary File SF1.2**.

To compare the difference between viral infection and cytokine stimulation, we infected ASH2–2 cells with ISAV and compared this response to that achieved by stimulating with rIFNg. Viruses such as influenza, another orthomyxovirus, encodes several proteins that actively shut off host gene expression (79). If ISAV uses similar approaches, comparing viral infection to interferon stimulation could detect such effects.

**Figure 2:**
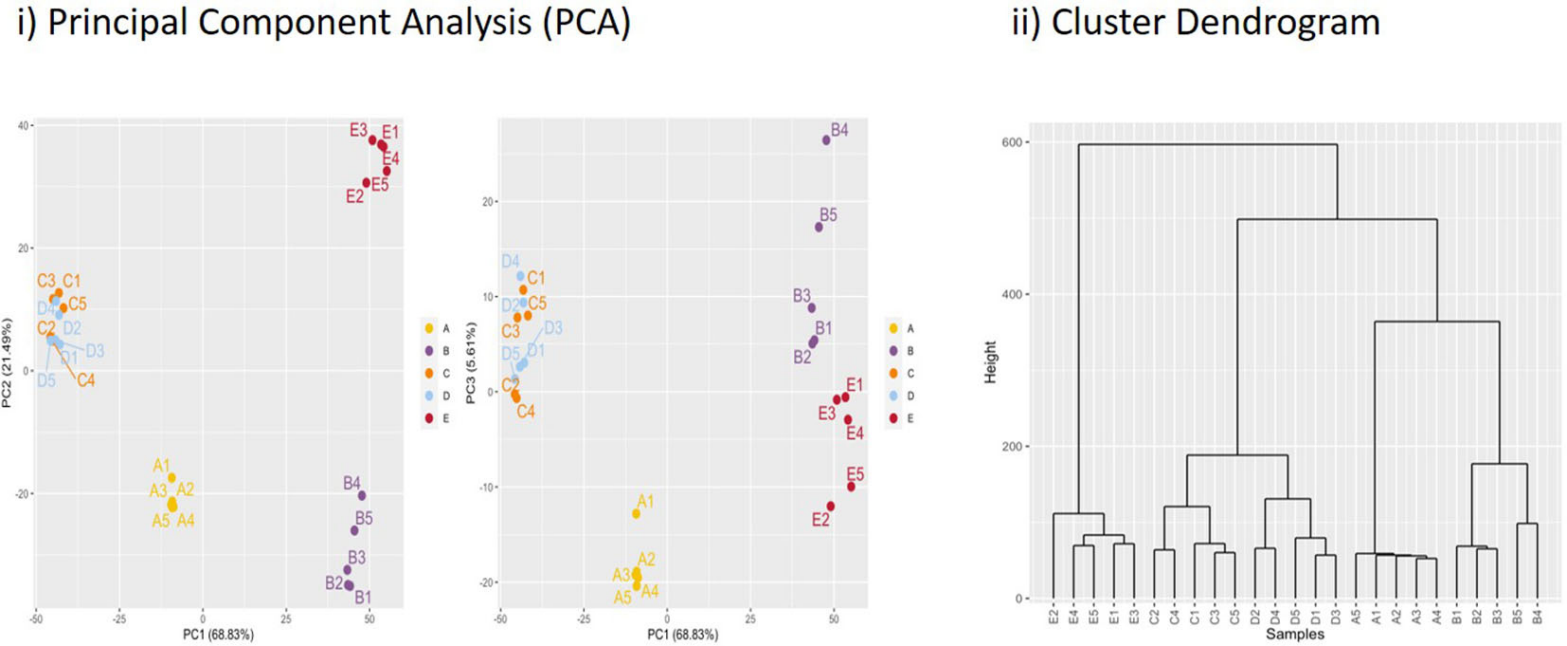
Principal component analysis (PCA) and hierarchical clustering dendrogram. (i) Principal component analysis for data from each replicate flask with percentages of variance associated with each axis. A1–5 (ISAV-4h) and B1–5 (ISAV-48h) are ISAV challenged samples, C (C-4h) and D (C-48h) are negative controls, while E is rIFNg-stimulated flasks (IFNg-48h). (ii) Sample clustering based on normalized data grouping replicates and separating biological conditions. A Euclidian distance is computed between samples and a dendrogram is built upon the Ward criterion.

Our sampling time points of 4 and 48 h for ISAV infection with similar negative control time points provided us with an overview of early and late responses. For comparison, we sampled interferon gamma-stimulated samples at 48 h post-stimulation (p.s.). Expression data for all differentially expressed genes for each biological replicate is shown in Supplementary file 2 (**Supplementary File SF2**). For each of the five samples we had five replicate flasks, i.e., ISAV-4h (A), ISAV-48h (B), negative control 4h (C), negative control 48h (D), and rIFNg-48h (E). All five replicates within each of the five A–E samples showed convincing clustering in principal component analysis and cluster dendrograms; thus, there was limited transcriptional variation between flasks (**Figure 2**; **Supplementary File SF1.2**). Expression profiles changed substantially from 4 to 48 h in the ISAV-challenged samples while a similar change was not observed in the negative control 4- to 48-h samples.

### 3.1 Viral sensors and early response genes

ISAV is a single-stranded RNA virus and should be recognized by various pattern recognition molecules. Based on the observed upregulation of viral sensors, the cell line responded well to infection even at 4 h p.i. despite fairly low expression of viral genes (**Table 2**; **Supplementary File SF1.3**). Four viral sensors display above 3.7 log2fold values after ISAV infection at 4 h, i.e., both the homeolog *DHX58* (alias *LGP2*) gene and the *RIG-1* (alias *DDX58*) gene and one of the homeolog *ZNFX1* genes. All these genes are more affected by virus than by rIFNg at 48 h. *MDA5* displays a similar upregulation in both virus and interferon samples at 48 h.

**Table 2:** Differentially expressed viral sensors and early response genes.

Gene name	Alias	ISAV-4h/C-4h	ISAV-48h/ISAV-4h	rIFNg-48h/C-48h
DExH-Box Helicase 58	DHX58a	4.97	2.31	6.19
	DHX58b	3.79	1.55	3.52
Viperin	RSAD2	6.19	5.62	8.93
Retinoic acid-inducible gene I	RIG-I	4.08	2.49	5.07
Zinc finger NFX1-type containing 1	ZNFX1a	−0.35	0.78	0.96
	ZNFX1b	4.30	1.42	4.77
Melanoma differentiation-associated protein 5	MDA5	0.81	1.57	2.69
Mitochondrial antiviral signaling protein	MAVSa	ns	−0.41	ns
	MAVSb	1.43	3.38	3.59
Interferon regulatory factor 3	IRF3	3.36	1.62	4.40
Interferon regulatory factor 7	IRF7.1	1.72	2.73	3.69
	IRF7.2	2.83	2.51	2.78
Myxovirus resistance protein	MX chr.12	4.04	3.66	5.60
	MX chr.25	1.59	5.59	15.39
Interferon-induced protein with tetratricopeptide repeats 8	IFIT8 chr.1	4.68	7.06	7.43
Interferon-induced protein with tetratricopeptide repeats 9	IFIT9 chr.28	5.34	3.14	5.23

Log2fold values between 2 and 4 are shaded pink, those between 4 and 10 are shaded blue, and those above 10 are shaded yellow. Individual read counts, genomic location, and gene IDs are shown in **Supplementary File SF1.3**, **SF2**. Not significant is shown using ns.

In mammals, both ZNFX1 and RIG-1 interact with MAVS. This MAVS activation results in the phosphorylation of interferon regulatory factors (IRFs) 3 and 7, which again induces expression of interferon and interferon-stimulated genes [reviewed in (80)]. In ASH2–2 cells, both the single *IRF3* and two of the three *IRF7* genes are more upregulated by virus than by rIFNg (**Table 2**). The third *IRF7* gene (ENSSSAG00000081805) residing on chr.10 displays no transcripts in our dataset. Of the duplicate *MAVS* genes, only the one on chr.9 responded with a log2fold increase of more than 3.3 upon ISAV-48h infection as well as rIFNg stimulation.

A comprehensive list of upregulated ISGs in both humans and a teleost is compiled in Levraud et al. (35). Some of these early responders are the myxovirus (*Mx*) and the IFN-induced protein with tetratrico-peptide repeats (*IFIT*) genes. Of the nine *Mx* genes described in Atlantic salmon, genes on chr.12 have been shown to respond more to type I IFN while genes on chr.25 are more affected by type II IFN (6, 81). This is also reflected in our model system where the gene on chr.25 responded more strongly to rIFNg while the gene on chr.12 responded more strongly to viral challenge with a log2fold value of 4 at 4 h p.i. (**Table 2**; **Supplementary File SF1.3**). *IFIT* genes have been implicated in various antiviral responses (82), and in our material, one gene residing on chr.1 and even more so one gene residing on chr.28 responded strongly to ISAV infection with log2fold values above 4.6 at 4 h p.i. (**Table 2**).

Both ISAV and rIFNg upregulated the transcription of many expected genes in ASH2–2 cells similar to what has been observed in other salmonid cell lines such as SHK-1, ASK, TO, or CHSE cells (69, 83–88). As for differentially expressed early response genes, several viral sensors are strongly affected by ISAV also at 4 h p.i. such as *RIG-I*, *LGP2*, and *MDA5* (**Table 2**). These three genes have previously been shown upregulated by various viruses or other stimulants in salmonids (69, 89, 90). Little data exist on *ZNFX1* in teleosts, but in humans, this gene is an early sensor of viral RNA (3), similar to what we see in our ISAV-4h samples.

In line with what has been described by Robertsen et al. (6), an *Mx* gene on chr.25 was more upregulated by rIFNg than the *Mx* genes on chr.12 while the gene on chr.12 was more affected by ISAV (**Table 2**). Owing to sequence divergence between the annotated Ensembl gene sequences and those of Robertsen et al. (6), a further definition of individual *Mx* genes in either region was not possible (data not shown).

### 3.2 Ubiquitin-like proteins

A master regulator induced by interferon or infection is *ISG15*, a ubiquitin-like protein attached to target proteins through the enzymatic cascade reaction called ISGylation [reviewed in (17)]. There are duplicate closely linked homeolog Atlantic salmon *ISG15* genes, two on chr.20 and four on chr.24 (**Table 3**; **Supplementary File SF1.4–1.6**) (77). One gene within each region is affected by virus with log2fold values above 4.5 at 4 h p.i. while the remaining *ISG15* genes are unaffected (**Table 3**; **Supplementary File SF1.6**). Of the other ubiquitin-like genes clustering with human *UBB*, *UBC*, *UBA52*, and *RPS27A* (**Supplementary File SF1**), none are significantly upregulated by virus or interferon (**Supplementary File SF1.7**).

**Table 3:** Differentially expressed ISG15 and related genes.

Gene name	Alias	ISAV-4h/C-4h	ISAV-48h/ISAV-4h	rIFNg-48h/C-48h
Interferon-stimulated gene 15	ISG15.1	4.56	6.04	7.56
	ISG15.4	5.81	6.49	10.73
Ubiquitin-activating enzyme E1 L5	UBE1.L5_	0.46	2.94	6.20
Tripartite motif containing 25	TRIM25a	0.28	0.94	0.50
	TRIM25b	2.27	1.09	2.13
Ring finger protein 213	RNF213a.1	0.88	4.65	2.20
	RNF213a.2	0.18	3.16	4.01
	RNF213a.3	1.82	2.78	3.98
	RNF213a.4a	0.88	1.79	2.48
	RNF213a.4b	1.36	1.66	2.20
	RNF213b	1.14	2.72	2.58
HECT and RLD domain-containing E3 ubiquitin protein ligases	HERC3a	ns	ns	ns
	HERC7a	6.14	3.36	7.05
	HERC9a	4.45	4.89	7.52
	HERC3b	ns	ns	ns
	HERC7b	5.33	2.67	6.60
	HERC9b	4.06	3.70	5.93
	HERC8	0.78	3.31	ns
Ankyrin repeat and IBR domain containing	ARIT.1	6.30	4.49	13.45
	ARIT.2	ns	4.29	11.59
Ubiquitin-specific peptidase 18	USP18a	3.08	0.85	3.92
	USP18b	3.13	0.59	3.96
	USP18-Like	1.99	2.26	4.93

Log2fold values between 2 and 4 are shaded pink, those between 4 and 10 are shaded blue, and those above 10 are shaded yellow. Individual read counts are shown in **Supplementary File SF1.6**, **SF2**. Not significant is shown using ns. Ensembl gene IDs and genomic location are shown in supplementary **Supplementary File SF1.6**.

The ISGylation process is mediated by the consecutive action of E1‐activating enzymes, E2‐conjugating enzymes, and E3 ligases that covalently link ISG15 to residues on target proteins (23). Only one E1‐activating enzyme gene displays log2fold values of 2.9 at 48 h p.i. and 6.2 at 48 h p.s. (*UBE1.L5*, **Table 3**; **Supplementary File SF1.6**). This gene is an ortholog to the human known ISG15 E1-activating enzyme UBE1L [ (16) and data not shown]. None of the annotated E2-conjugating enzyme genes display log2fold values above 2 in the infected or stimulated samples (data not shown).

In humans, ISG15 exhibits antiviral activity towards both DNA and RNA viruses, where, for instance, ISGylation of influenza A virus NS1 protein causes protein loss of function and thus inhibition of virus replication (17). Human ISG15 conjugation has been found essential for antiviral IFN response mediated by MDA5, a function that is counteracted by de-ISGylation from the papain-like protease of SARS-CoV-2 (91). Teleost *ISG15* has also been shown to be upregulated by a variety of viruses such as ISAV and IPNV in Atlantic salmon (21) and RGNNV in Asian seabass and black seabream (92, 93), supporting a role in antiviral defense also in teleosts. In Atlantic salmon, ISG15 and ubiquitin were shown covalently linked to segment 8 open reading frame 2 of the ISA virus (22). In humans, *ISG15* is primarily induced by type 1 interferons (16), while in teleosts, *ISG15* is induced by both type I and type II interferons [ (35) and this study]. Whether this difference also reflects downstream differences regarding function or interacting partners remains to be established as functional studies on ubiquitination or ISGylation in teleost are so far limited.

### 3.3 ISGylation

Attachment of Ub/L chains to proteins involves E1 ubiquitin-activating enzymes, E2 ubiquitin-binding enzymes, and E3 ubiquitin ligases. For human ISG15, the E1-activating enzyme is UBE1L (alias UBA7), and this is the only E1-activating enzyme shown to be affected by infection and interferon stimulation in our material (**Table 3**). Orthologs of the human ISG15 E2-conjugating enzyme UBCH8 (alias UBE2L6) were not significantly affected by virus or interferon gamma (data not shown).

E3 ubiquitin ligases provide the target specificity for adding ubiquitin or ubiquitin-like residues and are classified into RING, HECT, and RBR domain-containing E3 ligases [reviewed in (23)]. Of the E3 ubiquitin ligases known to be involved in ISGylation, TRIM25, HERC5, and ARIH1 belong to each of these three categories (16).

### 3.4 RING E3 ligases

TRIM molecules belong to the really interesting new genes or RING family (23). Based on sequence phylogeny, Atlantic salmon harbors two *TRIM25* homeologs located on chr.2 and chr.12 in addition to multiple *finTRIM* genes in which many are partial genes located on unplaced scaffolds (**Supplementary File SF1.8**). *TRIM25b* on the chr.12 gene displays a log2fold value above 2 in both the ISAV-4h and the rIFN-48h samples (**Table 3**; **Supplementary File SF1.6**). Only a few *finTRIMs* with known chromosomal location display log2fold values above 2.

In humans, TRIM25 is required for viral RNA sensing performed by the cytoplasmic helicase RIG-I, leading to IFN production (27, 94). *TRIM25* has also been implicated in antiviral immunity in teleosts, being a highly expressed ISG in studies of virus-infected cells or tissues in many species (32, 95, 96). In our material, we found one of the duplicate *TRIM25* orthologs to have log2fold values above 2 in both the ISAV-4h and the rIFN-48h samples (**Table 3**). Unfortunately, most of the finTRIMs are too fragmented in the investigated genome to allow a more detailed analysis of these genes.

Ring finger protein 213 (*RNF213*) also belongs to the RING family of E3 ubiquitin ligases and is upregulated by interferon stimulation and viral challenge in both humans and teleosts (34, 35). Humans only have one *RNF213* gene while two genes denoted *RNF213a* and *RNF213b* exist in zebrafish (97). We found six Atlantic salmon *RNF213* genes where five genes cluster with the zebrafish *RNF213a* sequence and one clusters with the zebrafish *RNF213b* sequence (**SF1.8**). Of the five *RNF213a* genes, one resides on chr.1, two on chr.3, one on chr.6, and one on chr.22 (**SF1.6**). All genes are more upregulated by virus at 48 h than by interferon with the gene *RNF213a.1* (ENSSSAG00000041408) on chr.1 displaying the strongest upregulation with a log2fold value of 4.6 (**Table 3**). One gene on chr.3 (*RNF213a.4a*, ENSSSAG00000043017) and the gene on chr.6 (*RNF213a.4b*, ENSSSAG00000047562) are homeologs (77) with a sequence identity of 98% (**Supplementary SF1.9**). The remaining *RNF213a* gene sequences share between 63% and 83% nucleotide sequence identity. A single Atlantic salmon *RNF213b* gene (ENSSSAG00000001848) located on chr.1 also displays log2fold values above 2.5 after 48 h p.i. as well as p.s. and shares 49–52 nucleotide sequence identity with the *RNF213a* sequences. In normal tissues, all salmon *RNF213* genes display low expression levels in immune-related tissues and negligible expression in non-immune tissues such as liver and heart, supporting immune gene function (**Supplementary SF1.10**).

Expression levels identified here may not be representative for all animals, tissues, or cell types. We thus chose to investigate the expression of *RNF213* genes in another Atlantic salmon cell line. SHK-1 is an Atlantic salmon cell line developed from head kidney in 1997 (98). This cell line was also defined as endothelial-like and was used to investigate differentially expressed genes following rIFNg stimulation using an old genome assembly from NCBI (GCF_000233375.1_ICSASG_v2) (69). Re-analyzing this dataset for DEGs identified in this study shows that three *RNF213a* genes in addition to the *RNF213b* gene were also upregulated in SHK-1, although less so than in the ASH2–2 cells (**Supplementary File SF1.11**). One gene (*RNF213a.1*) was not upregulated and *RNF213a.3* displayed no simple gene ortholog in the NCBI genome assembly (data not shown). Whether the differences between the two cell lines relate to individual or cell-specific differences remains to be established.

Human *RNF213* is upregulated by interferon and has been shown to act as a cellular sensor of ISGylated proteins with broad antimicrobial activity (18, 33). *RNF213* genes are also upregulated by stimulation or infection in other teleost species such as grouper or zebrafish (35, 99) suggesting an overall similar role as found for the human *RNF213* gene.

### 3.5 HECT E3 ligases

**Figure 3:**
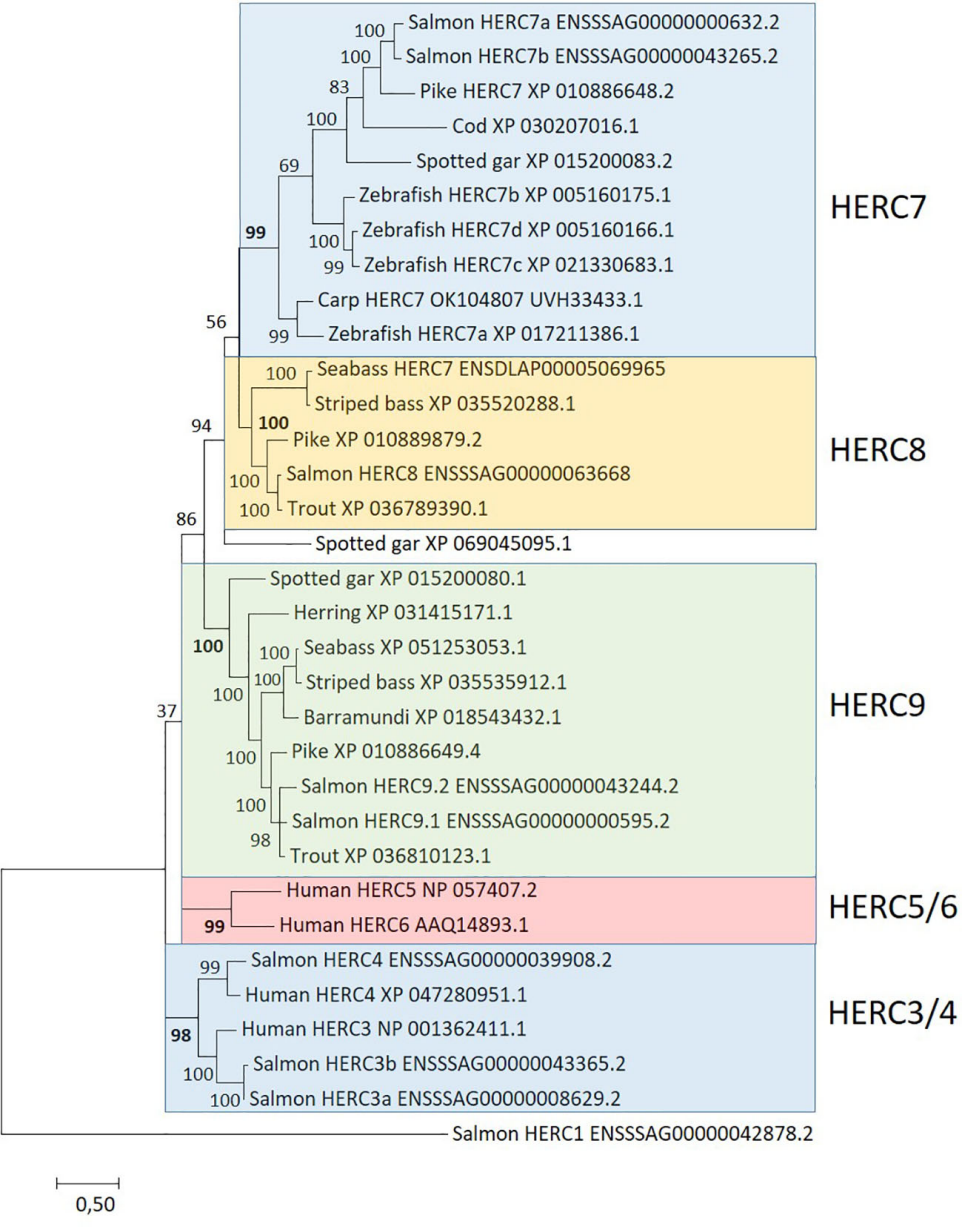
Phylogeny of deduced HERC amino acid sequences from salmon (*Salmo salar*), trout (*Oncorhynchus mykiss*), cod (*Gadus morhua*), striped bass (*Morone saxatilis*), seabass (*Dicentrarchus labrax*), barramundi (*Lates calcarifer*), herring (*Clupea harengus*), pike (*Esox lucius*), zebrafish (*Danio rerio*), spotted gar (*Lepisosteus oculatus*), and human. Sequence references are shown in the figure where *HERC7* sequences from carp, zebrafish, and seabass originate from (43, 44, 107), respectively. Clustering of sequences is highlighted using colored shading. The evolutionary history was inferred by using the maximum likelihood method based on the JTT matrix-based model (108). The tree with the highest log likelihood (−29,784.42) is shown. A discrete Gamma distribution was used to model evolutionary rate differences among sites (five categories [+G, parameter = 1.5507)]. The rate variation model allowed for some sites to be evolutionarily invariable [(+I), 1.29% sites]. The analysis involved 33 amino acid sequences. There were a total of 739 positions in the final dataset.

The second E3 ubiquitin-protein ligase family affected by ISAV and rIFNg is the HERC family. Based on blast search and sequence phylogeny, we found eight small Atlantic salmon HERC genes with two orthologs to human *HERC3* and one ortholog to human *HERC4* (**Figure 3**). Two salmon HERC gene sequences cluster with what has been defined as *HERC7* from carp and zebrafish (43, 44). The remaining three salmon HERC gene sequences reside in two other strongly supported clades. One clade includes only one sequence from salmon in addition to sequences from Northern pike and neoteleosts, which we here tentatively define as *HERC8* sequences. We could not find sequences from spotted gar or cyprinids belonging to this clade, suggesting that it may have arisen in a salmonid predecessor. The other clade includes two sequences from Atlantic salmon in addition to sequences from spotted gar, herring, and neoteleosts, here tentatively defined as *HERC9* sequences.

None of the three Atlantic salmon *HERC3* and *HERC4* genes were significantly affected by infection or stimulation (**Figure 3**; **Table 3**; **Supplementary File SF1.7**, **SF1.8**). On the other hand, all four *HERC7* and *HERC9* genes were strongly upregulated by ISAV with log2fold values above 4 in the ISAV-4h sample with increased transcript levels 48 h p.i. All four genes were also upregulated by interferon gamma, but less affected than by viral infection. The *HERC8* gene was only upregulated by ISAV at 48 h, but not affected by rIFNg. Looking at the transcription levels of these *HERC7–9* genes in normal tissues, the only gene strongly expressed in immune-related tissues is *HERC9a* with RPKM values above 50 in both head kidney and spleen and above 10 in gills (**Supplementary SF1.11**). Expression of the other four *HERC7–9* genes were low in immune-relevant tissues and negligible in non-immune organs. Based on sequence alignment and blast against NCBI TSA sequences, the salmon *HERC9b* gene may be a pseudogene expressing truncated transcripts (data not shown).

To verify expression in another Atlantic salmon sample, we again looked at expression in the SHK-1 cells stimulated with rIFNg (69). In addition, here there were similarities and differences between the two cell lines (**Supplementary File SF1.12**). Stimulatory effect on HERC3 and HERC4 genes was negligible in both cell lines. For HERC7a/HERC7b and HERC9a, upregulation was much higher in ASH2–2 cells than in SHK-1. These differences between the two cell lines may reflect cell-specific differences with ASH2–2 cells having a stronger response originating from a main target organ.

**Figure 4:**
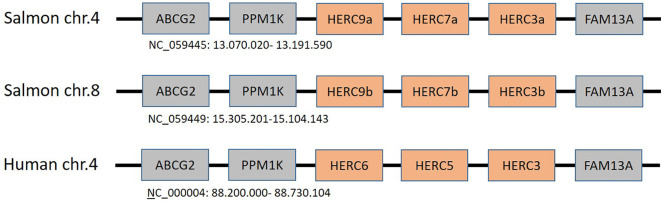
Regional synteny of genes surrounding the human *HERC5* and *HERC6* genes and the duplicate Atlantic salmon *HERC3*, *HERC7*, and *HERC9* genes on chromosomes chr.4 and chr.8. NCBI accession numbers with regional chromosomal coordinates are shown below each region.

*HERC5* and *HERC6* are defined as ISG15-specific E3 ubiquitin ligases with an antiviral effect on humans and mouse, respectively (35, 39–41). Teleosts were not thought to have direct orthologs to the mammalian *HERC5* and *HERC6*, but instead to have a unique teleost-specific sequence defined as *HERC7* that cluster with mammalian *HERC5* and *HERC6* gene sequences (43, 44) (**Figure 3**). However, this turns out not to be the case. In humans, dogs, and elephants, *HERC3*, *HERC5*, and *HERC6* genes reside closely linked on chr.4 in between *FAM13A* (family with sequence similarity 13 member A) and *PPM1K* (protein phosphatase, Mg^2+^/Mn^2+^-dependent 1K) genes whereas *HERC4* has translocated elsewhere (100). In Atlantic salmon, *c*losely linked *HERC3*, *HERC7*, and *HERC9* homeolog genes on chr.4 and 8 are also located in between *FAM13A* and *PPM1K* genes (**Figure 4**). This suggests that the salmon *HERC7* and *HERC9* genes evolved from their mammalian *HERC5* and *HERC6* counterparts. As in mammals, *HERC4* is located on a different chromosome (**Supplementary File SF1.7**).

Crucian carp *HERC7* has an E3 ligase activity for conjugation of both ubiquitin and ISG15 while zebrafish *HERC7c* only displays the potential to transfer ubiquitin (43, 44), while genes in both species are upregulated by viral infection. Based on the difference between the carp and zebrafish *HERC7* genes, we cannot predict if one or both salmon genes are involved in ISGylation. Whether salmon *HERC8* and *HERC9* are also involved in ISGylation will be determined by upcoming functional studies, but with large differences in expression levels of the duplicate *HERC9* genes in immune-related tissues, they most likely have different, as yet unknown functions. Being a species with many duplicate functional genes or homeologs following the fourth whole genome duplication, duplicate genes may evolve slightly different or entirely new functions (77).

### 3.6 RBR E3 ligases

**Figure 5:**
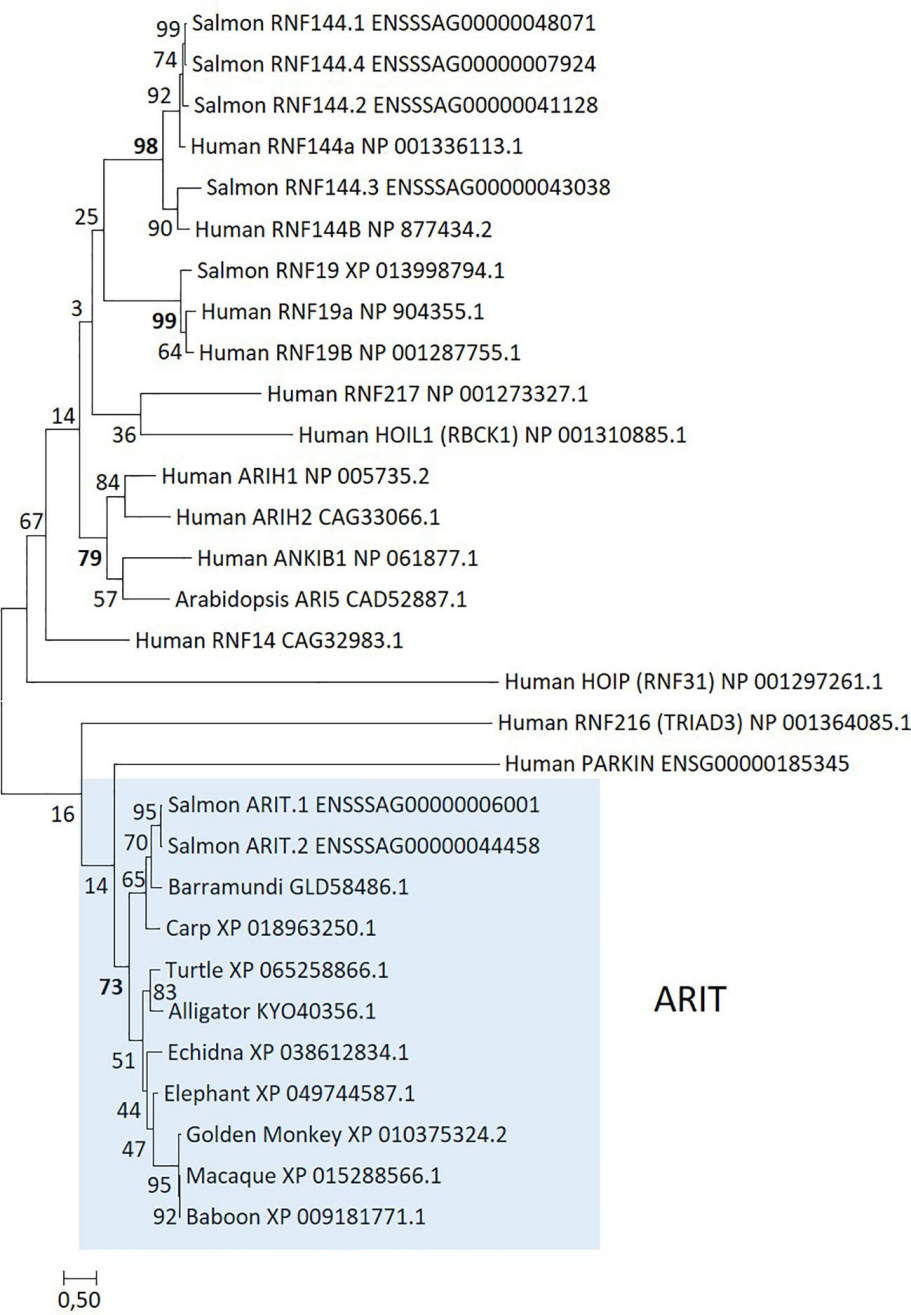
Phylogeny of deduced RBR family amino acid sequences from salmon (*Salmo salar*), arabidopsis (*Arabidopsis thaliana*), barramundi (*Lates japonicus*), carp (*Cyprinus carpio*), macaque (*Macaca fascicularis*), baboon (*Papio anubis*), golden snub-nosed monkey (*Rhinopithecus roxellana*), elephant (*Elephas maximus indicus*), echidna (*Tachyglossus aculeatus*), turtle (*Emys orbicularis*), alligator (*Alligator mississippiensis*), and human. Sequence references are shown in the figure and clustering of sequences is highlighted using colored shading. The evolutionary history was inferred by using the maximum likelihood method based on the Whelan and Goldman model (109). The tree with the highest log likelihood (−10,217.29) is shown. A discrete Gamma distribution was used to model evolutionary rate differences among sites [five categories (+G, parameter = 4.0718)]. The analysis involved 30 amino acid sequences. There were a total of 206 positions in the final dataset.

Although also a ring finger protein, *RNF144* belongs to the RBR family of E3 ubiquitin ligases. There are nine Atlantic salmon genes annotated as either *RNF144A* or *RNF144B* in the Ensembl genome where only two display significant upregulation by virus and even more upon interferon stimulation (ENSSSAG00000044458 and ENSSSAG00000006001). A blast search against other teleost sequences hit multiple teleost sequences annotated as *ARI5*, a gene only defined as an E3 ubiquitin ligase in arabidopsis (101). A phylogenetic analysis of these gene sequences showed that the two upregulated salmon genes do not cluster with other *RNF144* sequences or with *ARI5* from arabidopsis or any of the other defined RBR gene sequences (**Figure 5**). Instead, they form a unique and strongly supported cluster alongside sequences from teleosts, reptiles, and monkeys. A search for similar sequences in primates and humans turned out negative, so the gene has most likely been lost en route to hominoids. Sequences in this clade represent a currently undescribed member of the RBR E3 ligase family, containing the typical RBR domain structure. We tentatively suggest naming sequences belonging to this clade as *ARIT*, being first defined in a teleost. RBR E3 ubiquitin ligases share the RBR structure, but are classified into distinct subgroups based on 5’ and 3’ domains flanking the RBR domain. *ARIT* sequences only encode the RBR domain, with no additional 5’ or 3’ sequence, similar to the *RNF144* gene. However, *ARIT* sequences do not include a transmembrane domain as found in salmon and human *RNF144A* and *RNF144B* sequences [(102) and **Supplementary File SF1.12**].

Salmon *ARIT* sequences are strongly upregulated by rIFNg with log2fold values above 11 at 48 h p.s., but less so by ISAV infection with log2fold values above 4 at 48 h p.i. (**Table 3**; **Supplementary File SF1.6**). In unstimulated tissues, the *ARIT.1* gene displays low expression in immune-relevant tissues, but negligible expression in non-immune tissues (**Supplementary File SF1.10**). The salmon *ARIT.2* gene displays negligible expression in all tissues. Nucleotide sequence identity between the two salmon *ARIT* sequences is 98% (**Supplementary File SF1.9**); thus, some reads may have been misplaced for the two genes affecting their observed expression levels. None of the salmon *RNF144* sequences are significantly affected by virus or interferon (**Supplementary File SF1.7**).

We also investigated ARIT expression in the SHK-1 cells stimulated by rIFNg (69). Here, the log2fold values exceeded 7 in SHK-1 while they were above 11 in the ASH2–2 cells (**Supplementary File SF1.11**). Again, this could reflect differences in tissue origin for the two cell lines, as heart is a main target organ for the virus. Although not an ortholog, *ARIH1* has also been listed as an ISG15-specific E3 ubiquitin ligase in mammals (103). Whether the genes here defined as *ARIT* are ISG15-specific E3 ubiquitin ligases similar to the mammalian *ARIH1* gene needs further verification.

### 3.7 Deubiquitinases

Ubiquitin carboxyl-terminal hydrolases (USPs) assist in removing ubiquitin and ISG15 from proteins (54, 104). Only three of the annotated USPs in our material displayed a significant upregulation by infection or stimulation. In phylogenetic analyses, two of these DEGs cluster convincingly with human *USP18* (**SF1.13**), in mammals known to be specific for removing ISG15 moieties (54, 56). The third salmon DEG sequence clusters with sequences from tetrapods and teleosts mostly annotated as *USP47-like* and is a sister clade to the *USP18* sequences. Both the *USP4*7-like and the *USP18* clades are relatives of the human and teleost *USP47* sequences. We thus suggest introducing *USP18-like* as a nomenclature for the sequences belonging to the *USP47-like* clade. Homeolog salmon *USP18* genes display a log2fold upregulation above 3 in ISAV-4h as well as in rIFNg-48h samples (**Table 3**; **Supplementary File SF1.6**). The gene here defined as *USP18-like* displays a log2fold values above 2 in ISAV-48h samples, but above 4 in rIFNg-48h samples.

In humans, USP18 not only specifically de-conjugates ISG15 from target proteins, but also acts as a negative regulator of the type I interferon response (54, 104, 105). Whether salmon *USP18* and *USP18*-like genes have similar dual functions remains to be established.

### 3.8 Further processing of tagged proteins

When Ub/L molecules are attached by E3 ligases to proteins destined for degradation, they are processed by the 26S proteasome and some suitable fragments will be presented on the cell surface by MHC molecules.

The 26S proteasome complex consists of a 19S lid that in mammals contain multiple binding sites for ubiquitin or ubiquitin-like sequences. None of the salmon 19S subunits are significantly affected by infection or stimulation (data not shown).

In Atlantic salmon, the 20S core subunits denoted *PSMB8*, *PSMB9*, *PSMB10*, *PSMB12*, and *PSMB13* all reside within the duplicate MHCI regions on chr.14 and chr.27 (53). Of these, *PSMB10*, *PSMB12*, and *PSMB13* on chr.27 and *PSMB9*, *PSMB10*, and *PSMB12* on chr.14 display log2fold values above 2 in the rIFN-48h samples (**Table 4**; **Supplementary File SF1.15**). In the ISAV-48 samples, only *PSMB10* on chr.27 and *PSMB12* on chr.14 display log2fold values above 2. Three of the other 20S core subunits, not known to be inducible in mammals, are also affected by stimulation, i.e., *PSMA6.2*, *PSMA6.5*, and *PSMA7* with log2fold values above 2.8 in rIFNg-48 samples. *PSMA7.5* also displays a log2fold value of 2.5 in the ISAV-48h sample.

**Table 4:** Differentially expressed proteasome, MHC, and related genes.

	Gene	Alias	ISAV-4h/C-4h	ISAV-48h/ISAV-4h	rIFNg-48h/C-48h
MHCI (UDA) chr.14	Tapasin	TAPBPb	0.65	1.29	1.30
	Proteasome subunit beta type-8	PSMB8b	0.86	1.85	4.47
	Proteasome subunit beta type-12	PSMB12b	1.15	2.02	5.78
	Proteasome subunit beta type-9	PSMB9b	0.47	1.43	3.36
	Transport-associated protein 2	TAP2b	3.79	1.87	6.60
	Proteasome subunit beta type-10	PSMB10b	0.10	1.73	3.36
MHCI (UBA) chr.27	Tapasin	TAPBPa	1.78	1.19	5.53
	MHC class I	UBA	0.24	1.17	3.19
	Proteasome subunit beta type-13	PSMB13a	0.85	1.87	4.36
	Proteasome subunit beta type-12	PSMB12a	0.44	1.36	2.98
	Transport-associated protein 2	TAP2a	2.72	2.23	6.11
	Proteasome subunit beta type-10	PSMB10a	0.18	2.23	2.62
Other regions	Proteasome subunit beta type-6	PSMA6.2	0.56	1.74	3.75
		PSMA6.5	0.63	1.17	2.82
	Proteasome subunit beta type-7	PSMA7b	−1.36	2.51	3.76
	Transport-associated protein 1	TAP1	0.80	1.54	3.47
	Tapasin-related	TAPBPRb	ns	1,23	3.69
	Tapasin-like	TAPBPL1a	1.08	2.02	2.55
		TAPBPL2	1.37	2.09	3.90

Nomenclature adapted from Grimholt et al. (53). Genes residing within each of the duplicate MHCI regions on chromosomes 14 and 27 are shown on the left-hand side. Log2fold values between 2 and 4 are shaded pink while those above 4 are shaded blue. Individual read counts, Ensembl gene IDs, and genomic location are shown in **Supplementary File SF1.15**. Not significant is shown using ns.

Degraded MHC-compatible protein fragments are selectively transported into the endoplasmic reticulum (ER) through the TAP1 TAP2 channel. Duplicate *TAP2* genes residing on chr.14 and 27 are upregulated by both virus and rIFNg (**Table 4**, **SF1.15**). Salmon *TAPBP* on chr.27 is strongly upregulated by rIFNg with a log2fold value of 5.5 in the rIFNg-48h samples. Two of the *TAPBPL* genes also display log2fold values above 2 at 48 h in both the virus and the rIFNg samples. In a previous study, we only found one *TAPBPR* on chr.2 to be expressed and upregulated by stimulation (69). In the current Ensembl genome assembly, a homeolog on chr.2 lacks transcripts while the duplicate on chr.5 is strongly upregulated by rIFNg. Whether this difference relates to genome assembly issues or is a true difference between cell lines remains to be established.

Peptides transported into the ER and loaded into the MHCI groove are transported to the cell surface for recognition by T cells. UBA, a single classical MHCI gene as the main peptide presenter, displays a relatively high expression level also in unstimulated cells, but reaches a log2fold value above 3 in the rIFNg-stimulated sample (**Table 4**; **Supplementary File SF1.15**).

*PSMB10* and *PSMB12* display log2fold values above 2 in both regions while only *PSMB8* and *PSMB9* on chr.14 display a similar upregulation (**Table 4**). *PSMB10* is located closer to non-classical Z lineage genes and may not affect classical *UBA* gene peptides. Surprisingly, two *PSMA6* genes in addition to one PSMA7 gene were also upregulated by both rIFNg and ISAV. In humans, *PSMA6* has been shown to be upregulated during viral hepatitis, with a negative impact on *ISG15* gene expression (106), but no such data exist for human *PSMB7*. The salmon *PSMA6.2* gene was also upregulated by rIFNg in the SHK-1 cell line (69), suggesting that it may be a general effect in endothelial cells. Atlantic salmon has five expressed copies of the *PSMA6* gene, allowing some to diverge into new functions.

Of the MHCI and proteasome pathway genes, only *TAP2* in both duplicated regions displayed log2fold values above 2 at 4 h p.i. with a stronger upregulation by rIFNg at 48 h as compared to ISAV-48h. This may suggest viral interference with MHCI proteasome genes. However, another explanation could be that the transcription machinery is overtaken by viral transcripts. With the single exception of *RNF213a.1*, expression of all genes shown in **Tables 2**-**4** is higher in the rIFNg-48h sample than in the ISAV-48h sample. This suggests that the host transcriptional machinery shares its capacity between host and viral transcripts.

## 4 Conclusion

In mammals, *ISG15* is a key participant in the host antiviral response through a process called ISGylation where E1-activating enzymes, E2-conjugating enzymes, and E3 ligases attach the UbL chain to molecules to be functionally modified or destined for degradation. Here, we use an endothelial-like cell line originating from a main ISAV target organ to investigate how infection affects expression of genes in this ubiquitin–proteasome pathway. We show that in Atlantic salmon as in mammals, many ortholog genes are upregulated upon viral infection such as *ISG15*, *UBE1L*, *USP18*, and *TRIM25*. Atlantic salmon, like several other teleosts, have expanded on the human HERC5 and HERC6 genes where we add *HERC8* and *HERC9* genes to what was previously defined as teleost-specific *HERC7* genes. The salmonid-specific whole genome duplication event provided Atlantic salmon with duplicate *HERC7* and *HERC9* genes that are both strongly upregulated by virus even at 4 h after infection. The HERC8 gene is turned on at 4 h p.i. but quickly downregulated at 48 h p.i. The mammalian *ARIH1* gene seems absent from salmon, but we identified a cluster of sequences from both tetrapods and teleosts that we here denote *ARIT* genes. This gene is present in monkeys, but lost en route to hominids. For Atlantic salmon, one *ARIT* gene responds strongly at 4 h p.i., while both duplicates are upregulated at 48 h p.i. Functional studies of individual new E3 ubiquitin ligase molecules are needed to understand their biological relevance.

## Data Availability

The datasets presented in this study can be found in online repositories. The names of the repository/repositories and accession number(s) can be found below: https://www.ncbi.nlm.nih.gov/, PRJNA1197162.
