# Supplementary material for: ISGylation and E3 ubiquitin ligases: an Atlantic salmon genetic perspective
Source: Front Immunol. 2025 Jun 24;16:1554680. doi: 10.3389/fimmu.2025.1554680 (PMC12234341; doi:10.3389/fimmu.2025.1554680)
Supplement: Supplementary file 1 [file DataSheet1.pdf]

Supplementary File (SF) 1

Table of Content

		Page
SF1.1	Expression of Endothelial-specific markers in ASH2-2 cells	2
SF1.2	ISAV expression values (FPKM) in five replicate flasks at 4h and 48h timepoints	3
SF1.3	Differentially expressed Viral sensors and early response genes	4
SF1.4	Phylogeny of deduced Ubiquitin and Ubiquitin-like amino acid sequences	5
SF1.5	Alignment of deduced ISG15 sequences	6
SF1.6	Differentially expressed ISG15 and related genes	7
SF1.7	Other relevant differentially expressed genes	8
SF1.8	Phylogeny of selected deduced RNF213 amino acid sequences	9
SF1.9	Percent nucleotide sequence identity for RNF213, HERC and ARIT genes	10
SF1.10	Expression values (RPKM) for selected genes in unstimulated tissues	11
SF1.11	Log2fold values for selected rIFNG stimulated genes in SHK-1 and ASH2-2 cells	12
SF1.12	Alignment of selected deduced ARIT and RNF144 amino acid sequences	12
SF1.13	Phylogeny of selected deduced deubiquitinase sequences	15
SF1.14	Alignment of deduced USP18 sequences	16
SF1.15	Differentially expressed Proteasome, MHC and Peptide loading genes	17
SF1.16	References	17

SF1.1. Expression of Endotelial-spesific markers in ASH2-2 cells

		A	B	C	D	E	
Gene	Ensembl Gene ID	ISAV-4h	ISAV-48h	C-4h	C-48h	IFNg-48h	Gene description
CDH5	ENSSSAG00000041358	3237	5258	4353	4907	4506	cadherin-5-like
KDRL	ENSSSAG00000046825	2890	2893	3078	2920	2376	vascular endothelial growth factor receptor kdr-like
KDRL	ENSSSAG00000054371	1337	943	1272	1129	936	vascular endothelial growth factor receptor kdr-like
KDR	ENSSSAG00000066215	2843	2923	2847	3414	2609	kinase insert domain receptor
DUSP5	ENSSSAG00000007089	2750	1820	1671	2458	2520	dual specificity protein phosphatase 5-like
DUSP5	ENSSSAG00000027885	5217	3741	3102	4007	4531	dual specificity protein phosphatase 5-like
EDF1	ENSSSAG00000041563	1272	1394	1556	1463	1293	endothelial differentiation-related factor 1 homolog
EDF1	ENSSSAG00000057783	331	168	263	183	111	endothelial differentiation-related factor 1 homolog
EDF1	ENSSSAG00000068977	2216	2468	1996	2631	3318	endothelial differentiation-related factor 1-1
LIPG	ENSSSAG00000053486	673	1510	686	1388	1573	endothelial lipase-like
EPAS1	ENSSSAG00000030127	695	451	955	627	268	endothelial PAS domain protein 1
EDN2	ENSSSAG00000007155	443	206	453	260	223	endothelin 2
EDNRB-L	ENSSSAG00000001494	217	431	254	281	410	endothelin B receptor-like
EDNRB-L	ENSSSAG00000050997	208	629	277	390	1009	endothelin B receptor-like
ECE2A	ENSSSAG00000066389	119	105	134	110	189	endothelin converting enzyme 2a
EDN2	ENSSSAG00000046059	315	96	271	194	94	endothelin-2-like
ECE1	ENSSSAG00000004876	118	150	150	143	203	endothelin-converting enzyme 1-like
ECE1	ENSSSAG00000081046	596	561	619	730	1033	endothelin-converting enzyme 1-like
DDR1	ENSSSAG00000017278	421	241	499	411	666	epithelial discoidin domain-containing receptor 1-like
DDR1	ENSSSAG00000055704	128	74	96	166	272	epithelial discoidin domain-containing receptor 1-like
EMP2	ENSSSAG00000065265	598	762	1040	816	568	epithelial membrane protein 2
EMP1	ENSSSAG00000000360	12243	10672	21002	16499	4432	epithelial membrane protein 2-like
EMP1	ENSSSAG00000002465	101	179	130	262	189	epithelial membrane protein 2-like
EMP1	ENSSSAG00000007154	305	406	387	486	568	epithelial membrane protein 2-like
EMP1	ENSSSAG00000059678	6922	9516	13166	14356	5028	epithelial membrane protein 2-like
EMP3	ENSSSAG00000068369	359	813	530	513	268	Epithelial membrane protein 3

Column A-E show number of reads matching each gene within each sample representing cells from five individual 25 cm^2 ASH2-2 flasks. A and B are ISAV infected samples taken at 4 and 48 hours, C and D are negative controls taken at 4 and 48 hours, while E is rIFNg stimulated samples taken at 48 hours.

SF1.2. ISAV expression values (FPKM) in five replicate flasks at 4h and 48h timepoints

	ISAV-4h samples					ISAV-48h samples					Accession #	Length bp
	A1	A2	A3	A4	A5	B1	B2	B3	B4	B5		
S1_PB2	4117,11	4095,35	4294,68	4346,69	2795,12	70081,24	74797,99	68877,93	63427,07	79673,42	AY373381.1	2170
S2_PB1	3909,14	2821,57	3568,02	3286,41	3101,05	50278,09	52886,91	49150,85	47315,60	57395,97	AF404346.1	2126
S3_NP	1624,00	1302,43	1482,29	1706,62	1288,29	101161,97	91084,13	105823,37	157795,90	141255,82	AF404345.1	2047
S4_PA	116,20	0,00	0,00	195,38	0,00	1364,24	1613,98	1472,30	765,81	1612,27	AF404344.1	1788
S5_F	0,00	0,00	0,00	0,00	0,00	117,74	171,53	102,04	88,35	39,89	AF404343.2	1475
S6_HE	11944,45	8570,96	9508,65	8456,16	7779,74	398093,09	400083,20	393485,05	325192,16	307913,45	AF404342.1	1322
S7_Orf1	429,72	229,75	0,00	0,00	0,00	1127,19	1428,39	1291,12	1166,80	391,40	AF404341.1	967
S8_M	0,00	0,00	0,00	0,00	0,00	326,28	461,79	297,15	186,59	105,55	AF404340.1	737

Expression values of eight ISAV genes with accession numbers (Clotier et al., 2002) in each of the five replicate flask included in the ISAV-4h and ISAV-48h samples. Only open reading frame sequences shown with number of basepairs (bp) were used to calculate FPKM (Fragments Per Kilobase of transcript per Million mapped reads) values. S1 is segment 1 (S1) of the polymerase PB2, S2 is the polymerase PB1, S3 is the nucleoprotein NP, segment 4 is the polymerase acidic protein PA, S5 is the fusion protein F, S6 is the hemagglutinin-esterase HE, S7 encode the non-structural Orf1 protein NS1 while S8 encodes the matrix protein M.

Heatmap showing expression of viral ISAV genes per replicate flask:

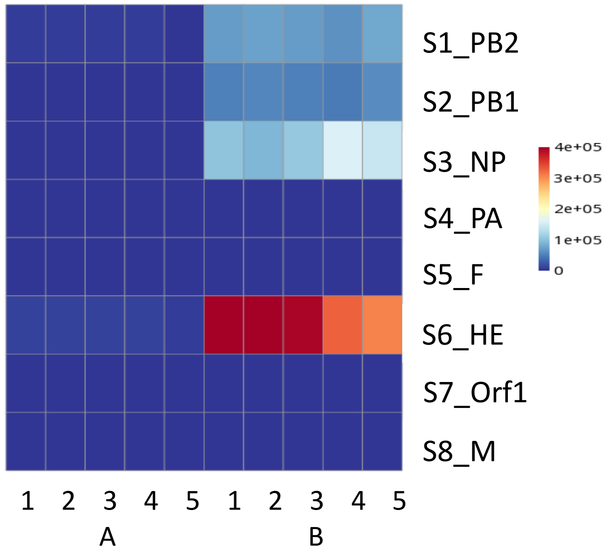

Heatmap was created based on the data in SF1.2 (https://gexijin.github.io/datamap/) using average linkage and Euclidean distance with no data transformation.

SF1.3. Differentially expressed Viral sensors and early response genes

		A	B	C	D	E	
**Gene**	**Ensembl ID**	**ISAV-4h**	**ISAV-48h**	**C-4h**	**C-48h**	**IFNg-48h**	**Genome location**
DHX58a	ENSSSAG00000003156	3689	18266	118	94	6764	chr.3:60.694.673-60.700.347
DHX58b	ENSSSAG00000037858	3058	8970	221	189	2171	chr.6:41.032.665-41.037.730
RIG-I	ENSSSAG00000045391	406	2274	24	25	811	CAJNNT020003670.1:167.158
ZNFX1a	ENSSSAG00000080227	566	970	723	648	1260	chr.12:44.230.621-44.255.525
ZNFX1b	ENSSSAG00000043036	6188	16587	314	168	4543	chr.22:51.200.095:51.216.435
MDA5	ENSSSAG00000078885	3402	10107	1941	1576	10161	chr.21:15.076.401-15.096.281
MAVSa	ENSSSAG00000000057	630	474	662	644	454	chr.1: 912.598-922.263
MAVSb	ENSSSAG00000063287	218	2269	81	73	877	chr.9:51.006.123-51.023.136
IRF3	ENSSSAG00000047292	1253	3856	122	129	2721	chr.19: 66.523.538-66.548.883
IRF7.1	ENSSSAG00000076373	876	5830	266	300	3859	chr.16: 21.989.153-21.999.864
IRF7.2	ENSSSAG00000075674	234	1338	33	30	215	chr.17: 1.194.336-1.202.559
MX chr.12	ENSSSAG00000077530	2435	30751	148	123	5987	chr.12:74.981.113-74.988.212
MX chr.25	ENSSSAG00000051775	6	306	2	0	7756	chr.25:48.057.657-48.109.560
IFIT8 chr.1	ENSSSAG00000000844	128	17187	5	3	460	chr.1:74.319.363-74.327.358
IFIT9 chr.28	ENSSSAG00000068365	7662	67312	189	128	4810	chr.28:6.948.766-6.965.414

Average number of reads across all five replicates for each of the five A-E groups are shown for each gene alongside Ensembl gene ID and genomic location.

SF1.4. Phylogeny of deduced Ubiquitin and Ubiquitin-like amino acid sequences

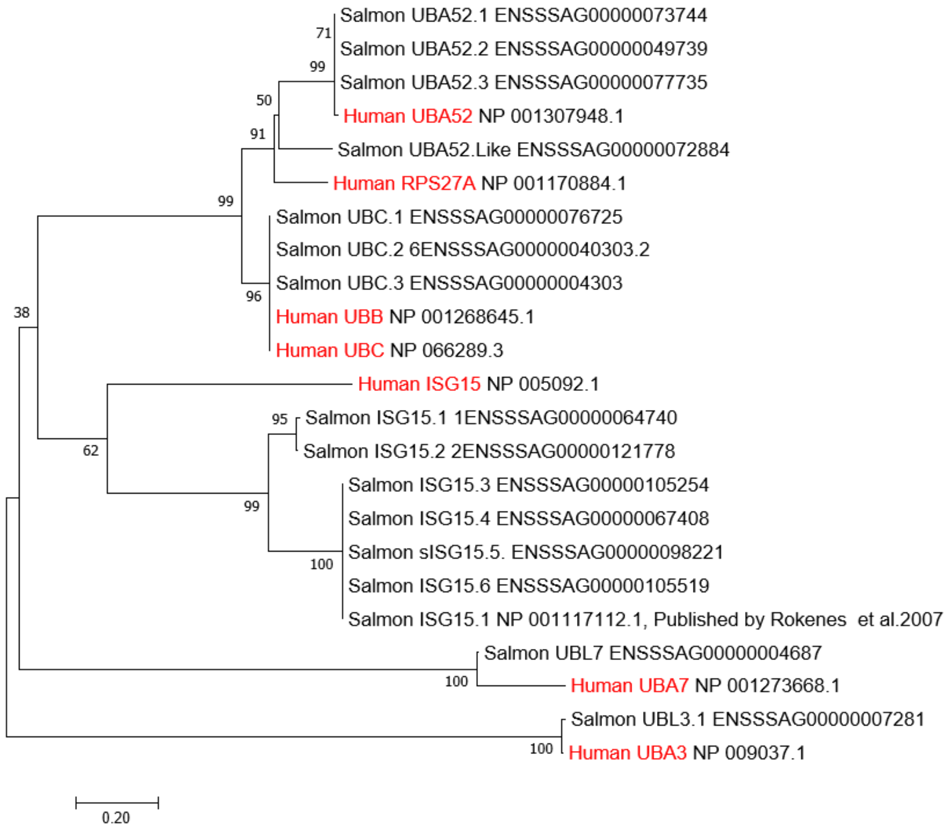

Legend to SF1.4: The evolutionary history was inferred using the Neighbor-Joining method (Saitou & Nei 1987). The optimal tree with the sum of branch length = 5.28203245 is shown. The percentage of replicate trees in which the associated taxa clustered together in the bootstrap test (1000 replicates) are shown next to the branches (Felsenstein 1985). The tree is drawn to scale, with branch lengths in the same units as those of the evolutionary distances used to infer the phylogenetic tree. The evolutionary distances were computed using the Poisson correction method (Zuckerkandl & Pauling 1965) and are in the units of the number of amino acid substitutions per site. The analysis involved 23 amino acid sequences. All positions containing gaps and missing data were eliminated. There were a total of 97 positions in the final dataset. Evolutionary analyses were conducted in MEGA7 (Kumar et al.2016). The first Atlantic salmon ISG15 gene sequence published by Rokenes and co-workers in 2007 is included for comparison.

SF1.5. Alignment of deduced ISG15 amino acid sequences

```
                      *        20         *        40
ssISG15.1  : MSKFQPQPGSFARLHNKDFSTISTEKISVMELTITLLNGESRPLMVQPH :  49
ssISG15.2  : -----------------------------.................... :  20
ssISG15.3  : -----------------------------..........N.L..T.... :  20
ssISG15.4  : -----------------------------..........N.L..T.... :  20
ssISG15.5. : -----------------------------..........N.L..T.... :  20
ssISG15.6  : -----------------------------..........N.L..T.... :  20
drISG15    : -----------------------------.Q..VK..G.DVKR.E.SGD :  20
hsISG15    : ---------------------------MGWD..VKM.A.NEFQVSLSSS :  22
             *        60         *        80         *       1
ssISG15.1  : TTLGSLKSLIKQHFGVAMGRQRLSGVNGNNISLSDDSKTLSDYGLHSGS :  98
ssISG15.2  : ..................E.............................. :  69
ssISG15.3  : ..V.......EE..K..TTK...L....S.................... :  69
ssISG15.4  : ..V.......EE..K..TTK...L....S.................... :  69
ssISG15.5. : ..V.......EE..K..TTK...L....S.................... :  69
ssISG15.6  : ..V.......EE..K..TTK...L....S.................... :  69
drISG15    : A.V.I..QV.S.Y.N.PTFK.K..AE..QR...E.E.R...S...N.D. :  69
hsISG15    : MSVSE..AQ.T.KI..HAFQ...-A.HPSGVA.Q.-RVP.ASQ..GP.. :  69
             00         *       120         *       140
ssISG15.1  : KLMLLI-TEPTHIQVFLKNDKGQTHTYEVVSGETVTQFKAKVQNKEGVP : 146
ssISG15.2  : ......-.....................L.............E...... : 117
ssISG15.3  : .V.V..-...AP.......E.......D..P.................. : 117
ssISG15.4  : .V.V..-...AP.......E.......D..P.................. : 117
ssISG15.5. : .V.V..-...AP.......E.......D..P.................. : 117
ssISG15.6  : .V.V..-...AP.......E.......D..P.................. : 117
drISG15    : VV....T.N.GTF...V..E...VK..D.DAN...D.LQT.IYQ..R.. : 118
hsISG15    : TVL.VVDKCDEPLSILVR.N..RSS....RLTQ..AHL.QQ.SGL...Q : 118
               *       160         *       180         *
ssISG15.1  : ANQQRLIHQGKQLDDDKKLEDYGVRNLSTIHLTLRLRGG--------- : 185
ssISG15.2  : .......................................--------- : 156
ssISG15.3  : ........E.R..E.CQT..Y.NI..Q............--------- : 156
ssISG15.4  : ........E.R..E.CQT..Y.NI..Q............--------- : 156
ssISG15.5. : ........E.R..E.CQT..Y.NI..Q............--------- : 156
ssISG15.6  : ........E.R..E.CQT..Y.NI..Q............--------- : 156
drISG15    : KD.....FK.R..ESGM..Q..DITS.....M.......--------- : 157
hsISG15    : DDLFW.TFE..P.E.QLP.GE..LKP...VFMN......GTEPGGRS- : 165
```

There are 6 Atlantic salmon (ss) ISG15 genes annotated in the Ensembl genome, two on chr.20 and four on chr.24, with some overlap between annotated transcripts (see below). Two gene sequences are supported by reads in our dataset i.e. ssISG215.1 and ssISG15.4.
ISG15 genes on Chr.20:
ssISG15.1_chr.20:29.237.298-29.237.777 (ENSSSAG00000064740)
ssISG15.2_20:29.409.959-29.410.438 (ENSSSAG00000121778)
ISG15 genes on Chr.24:
ssISG15.3_24:15.916.349-15.916.828 (ENSSSAG00000105254),
ssISG15.4_24:15.925.134-15.925.613 (ENSSSAG00000105254, ENSSSAG00000067408),
ssISG15.5_24:15.933.909-15.934.388, (ENSSSAG00000105254,ENSSSAG00000067408,ENSSSAG00000098221)
ssISG15.6_24:15.942.682-15.943.161 (ENSSSAG00000105519)
Other sequence references not shown in table ST4 are zebrafish (dr) NP_001191098.1 first identified by Liu et al.(2002) and the human (hs) ISG15 sequence NP_005092.1.

<u>Genes flanking ISG15 in humans and Atlantic salmon:</u>

Human ISG15 region: PLEKHN1-PERM1-HES4- ISG15-AGRN-RNF223
A.salmon CHR.20 (300 KB) region:
USP30-ALKBH2-UDG-PXMP2-ISG15.1-RBM19-PLBD2-MORC2A-AIDB-NCLX-TPCN1-ISG15.2-IQCD-CFAP73-TBX5-PLEKHA2

A.salmon CHR.24 (100 KB) region: GATC-SFRS9-ISG15.3-ISG15.4-ISG15.5-ISG15.6-SV2-SSHLA

SF1.6. Differentially expressed ISG15 and related genes

		A	B	C	D	E	
**Gene**	**Ensembl ID**	**ISAV-4h**	**ISAV-48h**	**C-4h**	**C-58h**	**IFNg-48h**	**Genome location**
ISG15.1	ENSSSAG00000064740	47	3110	2	3	433	chr.20:29.237.088-29.243.438
ISG15.4	ENSSSAG00000067408	56	4978	1	0	307	chr.24: 15.923.274-15.934.971
UBE1.L5	ENSSSAG00000047891	224	1715	162	71	5215	chr.12:85.646.032-85.791.344
TRIM25a	ENSSSAG00000054152	848	1621	697	1073	1514	chr.2:54.447.232-54.473.108
TRIM25b	ENSSSAG00000046838	1492	3164	310	281	1224	chr.12:32.833.462-32.849.914
RNF213a.1	ENSSSAG00000041408	457	11468	248	275	1268	chr.22:50.021.775-50.080.932
RNF213a.2	ENSSSAG00000078539	463	4138	409	376	6073	chr.1:167.399.939-167.480.842
RNF213a.3	ENSSSAG00000054674	1377	9448	390	406	6420	chr.3:8.183.152-8.247.558
RNF213a.4a	ENSSSAG00000043017	534	1846	290	305	1706	chr.3: 86,263,563-86,307,913
RNF213a.4b	ENSSSAG00000047562	524	1650	204	214	984	chr.6:15.539.681-15.617.465
RNF213b	ENSSSAG00000001848	1032	6786	468	504	3023	chr.1:83.492.992-83.538.985
HERC3a	ENSSSAG00000008629	131	77	118	89	84	chr.4:13.060.434-13.106.014
HERC7a	ENSSSAG00000000632	774	7951	11	12	1612	chr.4:13.062.729-13.072.374
HERC9a	ENSSSAG00000000595	131	3812	6	2	417	chr.4:13.049.444-13.059.289
HERC3b	ENSSSAG00000043365	645	755	488	453	598	chr.8:15.183.503-15.216.647
HERC7b	ENSSSAG00000043265	321	2043	8	4	369	chr.8:15.217.664-15.233.985
HERC9b	ENSSSAG00000043244	150	1954	9	8	534	chr.8:15.234.633-15.254.299
HERC8	ENSSSAG00000063668	62	611	36	38	48	chr.4:23.514.165-23.519.497
ARIT.1	ENSSSAG00000044458	21	467	0	0	3022	chr.7:24.018.681-24.045.226
ARIT.2	ENSSSAG00000006001	1	11	0	0	555	chr.7:24.100.000-24.109.941
USP18a	ENSSSAG00000002526	253	457	30	29	438	chr.7:45.848.318-45.853.985
USP18b	ENSSSAG00000046065	1883	2833	215	158	2466	chr.17:61.440.912-61.446.093
USP18-Like	ENSSSAG00000005966	159	765	40	31	974	chr.7:23.855.978-23.861.969
UBR4	ENSSSAG00000041331	1529	2311	1287	1456	2360	chr.15:103.769.849-103.783.361

Average number of reads across all five replicates for each of the five groups are shown for each gene alongside Ensembl gene ID and genomic location.

SF1.7. Differential expression of other relevant genes

		A	B	C	D	E	A / C	B / A	E / D	Genome location
		ISAV-4h	ISAV-48h	K-4h	K-48h	IFNg-48h	Log2-fold	Log2-fold	Log2-fold	
ISG15.2	ENSSSAG00000121778	nd	nd	nd	nd	nd	-	-	-	chr.20:29.409.375-29.412.801
ISG15.3	ENSSSAG00000105254	nd	nd	nd	nd	nd	-	-	-	chr.24:15.915.075-15.935.869
UBC.2	ENSSSAG00000040303	7842	8434	4909	5248	13563	0.68	0,11	1.37	chr.11:70.146.376-70.149.338
UBC.3	ENSSSAG00000004303	2095	3665	2023	2910	5109	0,06	0.81	0.81	chr.13:77.338.673-77.342.153
UBC.1	ENSSSAG00000076725	2596	2716	1721	2395	3719	0.59	0.07	0.64	chr.24:15.923.274-15.934.971
UBA52.1	ENSSSAG00000077735	1406	2155	1750	1983	4314	-0.32	ns	1.12	chr.3:15.496.147-15.515.904
UBA52.2	ENSSSAG00000049739	3596	3793	3400	3703	6705	0.08	ns	ns	chr. 10:16.596.966-16.599.459
UBA52.3	ENSSSAG00000073744	556	697	646	732	1196	-0.22	ns	ns	chr.14:13.392.227-13.394.313
UBA52.Like	ENSSSAG00000072884	6762	6793	6740	5793	9144	ns	ns	ns	chr.16:43.271.894-43.286.391
UBL3	ENSSSAG00000007281	402	383	640	408	507	ns	ns	ns	chr.11:100.315.110-100.524.787
UBL7	ENSSSAG00000004687	216	221	174	216	186	ns	ns	ns	chr.11:16.857.803-16.865.338
finTRIM	ENSSSAG00000072074	185	439	95	120	386	0.96	1.25	1.69	chr.7:27.261.471-27.301.633
finTRIM	ENSSSAG00000062102	11	10	14	6	20	ns	ns	1.84	chr.18:72.398.701-72.424.654
finTRIM	ENSSSAG00000000519	497	1587	110	110	708	2.18	1.674	2.67	chr.1:157315140-157346535
finTRIM	ENSSSAG00000048561	51	242	12	15	128	2.06	2.23	3.09	CAJNNT020000983.1:11536-13070
finTRIM	ENSSSAG00000013599	81	248	10	11	101	3.06	1.60	3.15	CAJNNT020004193.1:269763:271262
HERC3a	ENSSSAG00000008629	131	77	118	89	84	ns	ns	ns	chr.4:13060434-13106014
HERC3b	ENSSSAG00000043365	645	755	488	453	598	ns	ns	0.4	chr.8:15183503-15216647
HERC4	ENSSSAG00000039908	1389	1935	1687	1628	1512	ns	0.48	ns	chr.18:7188287-7212472
HERC1	ENSSSAG00000042878	387	253	243	247	251	bs	ns	ns	chr.13:105414736-105508908:
RNF144A.1	ENSSSAG00000048071	34	42	23	33	43	ns	ns	ns	chr.1:15.582.943-15.649.532
RNF144A.2	ENSSSAG00000007924	42	17	43	7	8	ns	ns	ns	chr.9:10.549.056-10.630.298
RNF144A.3	ENSSSAG00000041128	328	342	418	307	257	ns	ns	ns	chr.15:30.730.975-30.799.180
RNF144B	ENSSSAG00000043038	49	50	34	36	58	ns	ns	0.70	chr.5:64.386.022-64.394.847

Average number of reads across all five replicates for each of the five groups are shown for each gene alongside Ensembl gene ID and genomic location. Log2fold expression values for selected comparisons are also shown where log2fold values above 2 are shown using red cells.

SF1.8. Phylogeny of selected deduced RNF213 amino acid sequences

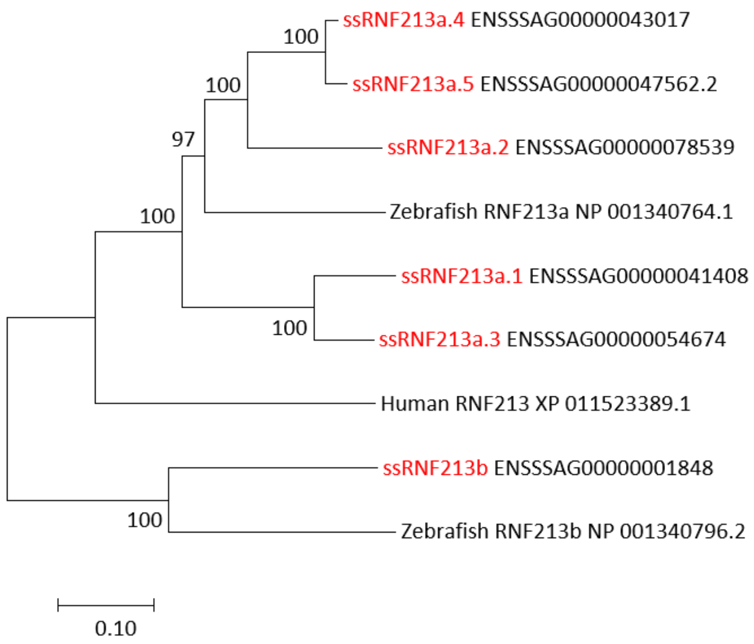

Legend to Figure SF1.8. The evolutionary history was inferred using the Neighbor-Joining method (Saitou N. and Nei M. (1987). The optimal tree with the sum of branch length = 1.88981736 is shown. The percentage of replicate trees in which the associated taxa clustered together in the bootstrap test (1000 replicates) are shown next to the branches (Felsenstein J. 1985). The tree is drawn to scale, with branch lengths in the same units as those of the evolutionary distances used to infer the phylogenetic tree. The evolutionary distances were computed using the Poisson correction method (Zuckerkandl E. and Pauling L. 1965) and are in the units of the number of amino acid substitutions per site. The analysis involved 9 amino acid sequences. All ambiguous positions were removed for each sequence pair. There were a total of 2799 positions in the final dataset. Evolutionary analyses were conducted in MEGA7 (Kumar S., Stecher G., and Tamura K. 2016).

SF1.9. Sequence identity of Atlantic salmon ARIT, HERC and RNF213 gene sequences

	ARIT.1	ARIT.2
ARIT.1		99
ARIT.2	99	

	HERC7.1	HERC7.2	HERC8	HERC9.1	HERC9.2
HERC7.1		92	57	47	51
HERC7.2	92		57	48	51
HERC8	57	57		67	75
HERC9.1	47	48	67		94
HERC9.2	51	51	75	94	

	RNF213a.4	RNF213a.5	RNF213a.2	RNF213a.3	RNF213a.1	RNF213b
RNF213a.4		98	77	69	64	50
RNF213a.5	98		77	69	63	50
RNF213a.2	77	77		67	63	49
RNF213a.3	69	69	67		83	52
RNF213a.1	64	63	63	83		49

Percent nucleotide sequence identity was calculated using Clustal X (Larkin et al.2007).

SF1.10. Expression (RPKM values) for selected genes in normal unstimulated tissues

Gene	HK	Gills	Gut	Spleen	Nose	Heart	Liver	Brain	Gene length (bp)	Ensembl ID
RNF213a.1	0,97	0,21	0,52	1,4	0,23	0,02	0,08	0,05	3057	ENSSSAT00000001095.2
RNF213a.2	1,53	0,85	0,83	1,73	0,29	0,08	0,07	0,12	1833	ENSSSAT00000170540.1
RNF213a.3	6,61	3,03	2,55	6,32	1,59	0,26	0,21	0,15	2955	ENSSSAT00000001153.2
RNF213a.4	5,09	3,13	2,39	4,08	1,41	0,33	0,5	0,7	1968	ENSSSAT00000001173.2
RNF213a.5	3,95	2,06	1,25	2,96	0,75	0,24	0,27	0,35	741	ENSSSAT00000171713.1
RNF213b	6,10	3,10	1,85	4,97	2,39	0,45	0,48	0,29	747	ENSSSAT00000013003.2
HERC9a	50,99	14,35	7,97	55,09	8,99	5,72	2,87	2,99	11202	ENSSSAT00000003995.2
HERC9b	2,67	1,01	0,99	3,26	1,69	0,66	0,18	0,4	16002	ENSSSAT00000209112.1
HERC8	1,11	0,30	0,18	1,28	0,43	0,13	0,18	0,25	16001	ENSSSAT00000184130.1
HERC7a	3,40	1,56	0,39	4,09	0,62	0,47	0,39	0,31	11940	ENSSSAT00000087520.2
HERC7b	7,22	2,43	1,38	7,18	1,73	0,64	0,28	0,64	15162	ENSSSAT00000197784.1
ARIT.1	2,27	4,39	1,70	4,34	1,82	0,16	0,16	0,2	11619	ENSSSAT00000059624.2
ARIT.2	0,71	0,18	0,14	1,1	0,23	0,05	0	0,05	2124	ENSSSAT00000063470.2
# reads	59084708	59793962	59806348	60203316	59545012	58163180	58784272	58939250		
SRR ID	1422860	1422858	1422859	1422870	1422867	1422862	1422865	1422856		

RPKM (Reads Per Kilobase per Million mapped reads) values for selected genes defined in tissue transcriptomes from head kidney (HK), gills, gut, spleen, nose, heart, liver and brain with Sequence Read identities shown (SRR ID). Read mapping was performed with a stringency of 98% identity and 95% coverage. Nucleotide sequence lengths used in the analysis is indicated in base pairs where only open reading frame sequences were included. RPKM values below 1 are shown using grey colored cells, those above 10 are shown using blue cell and those above 50 using orange cells. Ensembl transcript IDs used for the analysis is shown on the right hand side of the table.

SF1.11. Log2fold values for selected rIFNG stimulated genes in SHK-1 and ASH2-2 cells

Gene Alias	NCBI gene ID	Ensembl gene ID	SHK-1	ASH2-2
RNF213a.1	XM_014168095.1	ENSSSAG00000041408	0.15	2.20
RNF213a.2	XM_014145768.1	ENSSSAG00000078539	3.64	4.01
RNF213a.4a	XM_014194369.1	ENSSSAG00000043017	1.89	2.48
RNF213a.4b	XM_014194371.1	ENSSSAG00000047562.2	1.53	2.20
RNF213b	XM_014212626.1	ENSSSAG00000001848	1.42	2.58
HERC3a	XM_014194881.1	ENSSSAG00000008629	- 0.09	ns
HERC3b	XM_014209505.1	ENSSSAG00000043365	-0.73	0.4
HERC4	XM_014154277.1	ENSSSAG00000039908.2	ns	ns
HERC7a	XM_014209557.1	ENSSSAG00000000632	1.53	7.05
HERC7b	XM_014209558.1	ENSSSAG00000043265	1.03	6.60
HERC9a	XM_014194869.1	ENSSSAG00000000595	1.79	7.52
ARIT.1	XM_014185653.1	ENSSSAG00000044458	7.85	13.45
ARIT.2	NM_001141354.1	ENSSSAG00000006001	7.79	11.59

Log2fold values for selected 48 hours rIFNg stimulated genes in ASH2-2 cells (this study) and SHK-1 cells (Grimholt et al.2020). DEG analysis is SHK-1 cells was performed using the NCBI genome assembly GCF_000233375.1_ICSASG_v2 with NCBI gene accession numbers (XM_..) as opposed to the Ensembl genome (GCA_905237065.2) and gene references (ENSSSAG..) used in this study. The entire SHK-1 DEG dataset can be found in NCBI Sequence Read Archive BioProject accession number PRJNA637094. ns defines not significant.

SF1.12. Alignment of selected deduced ARIT and RNF144 amino acid sequences

```
                        *        20         *        40         *        60
SasaARIT.1   : -------------------MCAALD-----------DDTETLKAEMSCGHAVTPESLTGWCRS :  33
SasaARIT.2   : --------------MSNRHLI....-----------........................... :  38
Barramundi   : MTTRGEGEKRYDPRDTTLKFVNRP.DLD---PLPPEEGDRC.R.................... :  60
Carp         : MTEQQREEKRYDPKDKTLKFVTRQ.D------ITGD..P..RR...........Q...A.... :  57
Echidna      : -----MMSRPPLYSEPSPVFV.GK.E------ITGDEYNGV.RV........D.G...S.... :  52
Elephant     : ---MSHSKTEDFHNQRPLKFVLRK.D------ITGDEYDGQ.RV........D.G...A.... :  54
Turtle       : -----MEFFRKENEEPAPKFVRRK.D------ITGDEEE-LMRV.......AD.G........ :  51
Alligator    : --MSSFSPVPSKAEEPQLKFVRRK.D------ITGD.ED-IMRV.L.....TE.Q..AA.... :  54
Macaque      : --------MSHSAPEPPLKFVQGK.E------ITGDEYDGVDRV........D..N.KL..LQ :  49
Baboon       : --------MSHSAPEPPLKFVQGK.E------ITGDEYDGVDRV........D..N.KL..LQ :  49
GoldMonkey   : --------MSHSAPEPLLKFVQGK.E------ITGDEYDGVDRV........D..N.KL...Q :  49
SasaRNF144.1 : ----------MTTARYRPTWDL...PLVSCKLCLGEFPL.QMTTITQ.QCVFCTLC.KQYVEL :  53
SasaRNF144.2 : -------MTTGPSARYRPTWDL...PLMSCKLCLGEFPR.QMTTISQ.QCVFCSLC.KQYVEL :  56
SasaRNF144.3 : -------------------------------------------MPS.FCCCYSSWC.QQYVQL :  20
SasaRNF144.4 : ----------MTTARYRPTWDL...PLVSCKLCLGEFPL.QMTTITQ.QCVFCTLC.KQYVEL :  53
HumRNF144A   : ----------MTTTRYRPTWDL...PLVSCKLCLGEYPV.QMTTIAQ.QCIFCTLC.KQYVEL :  53
HumRNF144B   : MGSAGRLHYLAMTAENPTPGDL.PAPLITCKLCLCEQSLDKMTTLQE.QCIFCTAC.KQYMQL :  63
                                            <------------Ring-1---------------

                     *        80         *       100         *       120
```

```
SasaARIT.1   : LLDQG-QFKFLCPALEDGTFVKCGAEWSYQEVRRLAVLTTEEMEHFEKTMAALAAATYCEYKS :  95
SasaARIT.2   : .....-..............................................F---....... :  97
Barramundi   : .....-.Y..K....K...Q..D.V..............S...Q...ENI.R...IE...F.T : 122
Carp         : .....-.H.......KE..LQR.N...P.A.........Q..QS...E...V....E...H.T : 119
Echidna      : .....-YTT.H...V.GV--T....Q.P.T....H.S.NE..QHN..QKL.IT..QK..DF.Q : 112
Elephant     : .M...-H..LC...DIN.--E....Q.P.P...QC...NDT.QYQ..QKL.L...RS..DF.E : 114
Turtle       : .....-HL..H...DVN.--E...K....P....N.SI.EA.QRN..EKL.KF..KY.HD..E : 111
Alligator    : .....-Y.N.H...DVN.--Q...E........QN.L..E..Q.E...KLV.F..KY.SDL.E : 114
Macaque      : .IK..-KYTLH...EVK.--K....Q.L.P....CTQ.SDS.QQE..QGL.QA.MRR..NL.V : 109
Baboon       : .IK..-KYTLH...EVK.--K....Q.L.P....CTQ.SDS.QQE..QGL.QA.MRR..NL.V : 109
GoldMonkey   : .IK..-KYTLH...EVK.--K....Q.L.P....CTQ.SES.QQE..EGL.RA.MRH..NL.V : 109
SasaRNF144.1 : .IKE.LETAIS..DSACPK-RGHLL.NEIECMVASE.MQRHKKLQ..REVLLDPCL.W.PSS. : 115
SasaRNF144.2 : .IKE.LETTIS..DSACPK-QGHLL.NEIECMVAAGSMQRYRRLQ..REVLLDPCR.W.PSS. : 118
SasaRNF144.3 : AIRE.GGSPVT..DTACQR-TGVLLHSEIACFAPADQVELYQQLE..RGVQLDPSRAW.PVLE :  82
SasaRNF144.4 : .IKE.LETAIS..DSACPK-RGHLL.NEIECIVAT.IMQRYKKLQ..REVLLDPCR.W.PSS. : 115
HumRNF144A   : .IKE.LETAIS..DAACPK-QGHLQ.NEIECMVAAEIMQRYKKLQ..REVLFDPCR.W.PAST : 115
HumRNF144B   : AIRE.CGSPIT..DMVCLN-HGTLQ.AEIACLVPVDQFQLYQRLK..REVHLDPYR.W.PVAD : 125
               ---------------->                      <------------IBR---------

                  *         140         *         160         *         180
SasaARIT.1   : CPVCETFVEREDLTNLSVLCTVCTADKGQRYEFCWQCLKQWKGPGPRSDRCDNDGCINQDLEL : 158T
SasaARIT.2   : ............................................................... : 160
Barramundi   : ..G.K.Y.........N.Q..I.Q...KKV.Q.......P...KA..A.....N..V.H.... : 185
Carp         : ..G.QS....A.I...C.M..I...E..HAFQ.....MRE.............P..T.P.I.K : 182
Echidna      : ..G.KSL...K.....R....I.QVRR.KTF........P.T.A.TP..K.ADP..R.VKVDT : 175
Elephant     : ..S.KSL...KE..TIR.......STLRAT........RA...A.TP..H.A.V..KDPN..V : 177
Turtle       : ..N.HSY...Q..K..R.V.IL.HSL...GF........P...G.AP.E..E.V..K..S..V : 174
Alligator    : ..K.KS....K.....R.H.IL.LSL..EQF........P...S.TS.VK.A.V..R..S.DI : 177
Macaque      : ..G.RSL...K.PAE.R.H....CTIR.VP.D.....MQA....MLS.N..G.ED.RDPI.HI : 172
Baboon       : ..G.RSL...K.PAE.R.H....CTIR.VP.D.....MQA....MLS.N..G.ED.RDPI.HI : 172
GoldMonkey   : ..G.RSL...K.PVE.R.H....C.IR.VP.D.....MQA....MLS.N..G.KD.RDPI.HI : 172
SasaRNF144.1 : .QAVCQLK.T.VALPQL.Q.A...------L...SA.KAN.HP----GQA.PPPENNLPITAF : 168
SasaRNF144.2 : .QAVCQLK.G.VALAQL.Q.S..R------L...SA.QAS.HK----GQA.---QDNMPIITF : 168
SasaRNF144.3 : .QAVCSVGPSSEGQPT..P.LA.H------TV..SG.RGP.QD----GHA.P---EHQPMTSS : 132
SasaRNF144.4 : .QAVCQLK.M.VAQPQL.K.A...------L...SA.KAN.HP----GQV.PPQENNLPITAF : 168
HumRNF144A   : .QAVCQLQDVGLQ.PQP.Q.KA.R------M...ST.KAS.HP----GQG.P---ETMPIT-F : 164
HumRNF144B   : .QTVCPVASSDPGQPVL.E.PS.H------LK..SC.KDA.HA----EVS.R---DSQPIVLP : 175
               -------------------IBR---------------------------->

               *       200         *       220         *       240         *
SasaARIT.1   : LKNCETTSLPQVAGVTDCPSIRACPTCGQQVEHDKTGCKNILCPRCNKEFCFVCLKLTEDCLE : 221
SasaARIT.2   : .............................R................................. : 223
Barramundi   : ....KD......Q..DK............R...........I....QI..........PE..K : 248
Carp         : .AK.REIK..E.KN.S-...M.......NL....T......I.N..RV........V.QV..Q : 244
Echidna      : .A..SLKD..GSE-IKN............LI..LEK-..YV..SQ.QV....A..D.APA.RA : 236
Elephant     : .AS.T.KD..GSE-IRA...........LLI..KEK-..YVV.S..QV....A..E.ARA.QA : 238
Turtle       : .A..KLKD..GSE-IKN...........RLI..MEK-..Y.V..Q.HV....A..EIARN.QA : 235
Alligator    : .A..KLKP..GSE-IR............RLI..KEK-..YVM.TQ.HV....A..ETAQE.QA : 238
Macaque      : .AT.A.KD..DSS-IQG........E..LLI..KER-..YVT.S..DTK...A..ETAQA.EA : 233
Baboon       : .AT.A.KD..DSS-IQG........E..LLI..KER-..YVT.S..DTK...A..ETAQA.EA : 233
GoldMonkey   : .AT.A.KD..DSS-IQA........E..LLI..KER-..YVT.S..DTK...A..ETAQA.EA : 233
SasaRNF144.1 : .PGETSSFYKSDD---.DGP.KR..K.KVYI.R.EG-.AQMM.KN.KHA..WY..ESLD.DFL : 227
SasaRNF144.2 : .PGESSSFYKSDD---.DAP.KC..K.KVYI.R.EG-.AQMM.KN.KHA..WY..ESLD.DFL : 227
SasaRNF144.3 : SPAS.SRGRSCSD---SDLP.KQ..M..VYI.RNQG-.AQM..KS.KHT..WY..QNLDGDIF : 191
SasaRNF144.4 : .PGETSSFYKSDD---.DGP.KR..K.KVYI.R.EG-.AQMM.KN.KHA..WY..ESLD.DFL : 227
HumRNF144A   : .PGETSAAFKMEE---.DAP.KR..K.KVYI.R.EG-.AQMM.KN.KHA..WY..ESLD.DFL : 223
HumRNF144B   : ---T.HRA.FGTD---AEAP.KQ..V.RVYI.RNEG-.AQMM.KN.KHT..WY..QNLDNDIF : 231
                                      <-------Ring-2---------------->
```

```
                     260         *        280         *        300         *
SasaARIT.1   : TS--SYFTACSAGVAPRQTSIPTWKRN------------------------------------ : 246
SasaARIT.2   : ..--.......................------------------------------------ : 248
Barramundi   : ..--...IP..T..........V.H..------------------------------------ : 273
Carp         : ..--N..IG..D..........S.N..------------------------------------ : 269
Echidna      : SKAGAW.KY.AKPL.....K..V.SQRNRQKSP------------------------------ : 269
Elephant     : .KAGAW.KC.AKPL.....H..V.S.QGGGLWGDEVNTGHREQSHTPTV-------------- : 287
Turtle       : SKRGA..QY.AKPL..K..Q..V.AQK------------------------------------ : 262
Alligator    : KKHGT..LY.TKP......T..V.S-RK----------------------------------- : 265
Macaque      : AKPA.W.KW.AKPL....SHV.V.SPYQQRLLLQGQPLELEAFRPRAVFAQQPAANEERCLIL : 296
Baboon       : AKPA.W.KR.AKPL....SHV.V.SPYQQRLLLQGQPLELEAFQPRAVFAQQPAANEGRCLIL : 296
GoldMonkey   : AKPA.W.KW.AKPL....SHV.V.SPYQQRLLLQGQPLELEAFQPRVVFAQQPAANEGRCLIL : 296
SasaRNF144.1 : LI--HFDKGPCQNKLGHSR.SVI.H.TQSLTLLLSLLPSPPLSLSLLLFFLLLSSLALTLSRW : 288
SasaRNF144.2 : LI--H.DKGPCRNKLGHSRASVI.H.TQVVGIFAGFGLLLLVASPFLLLA------------- : 275
SasaRNF144.3 : LR--H.DKGPCRNMLGHSRASVM.N.TQVVGILVGVSVIVLVASPLLLLA------------- : 239
SasaRNF144.4 : LI--H.DKGPCRNKLGHSRASVI.H.TQVVGIFAGFGLLLLVASPFLLLA------------- : 275
HumRNF144A   : LI--H.DKGPCRNKLGHSRASVI.H.TQVVGIFAGFGLLLLVASPFLLLA------------- : 271
HumRNF144B   : LR--H.DKGPCRNKLGHSRASVM.N.TQVVGILVGLGIIALVTSPLLLLA------------- : 279
                                          < Transmembrane domain >

                  320         *        340         *        360
SasaRNF144.1 : WGSSLALACSCWWPLPSSSWPHHSSSAASASAAKEMMTPFPPKPHPTNTGG : 339
SasaRNF144.2 : --TPFVLCCKCKCSKGDDDPLPT---------------------------- : 296
SasaRNF144.3 : --SPCILCCLCKPCRGKKKRRK--KKELTQTDIQPELSPTKS--------- : 277
SasaRNF144.4 : --TPFVLCC------------------------------------------ : 282
HumRNF144A   : --TPFVLCCKCKCSKGDDDPLPT---------------------------- : 292
HumRNF144B   : --SPCIICCVCKSCRGKKKKHDPSTT------------------------- : 303
```

Alignment was perform using clustal X (Larkin et al.2007) and visualised using GeneDoc. Amino acids are color coded according to physiochemical properties. Empty lines are not shown. Sasa is *Salmo salar* and Hum is human where latin names and sequence references can be found in main text Figure 5. Individual domains, Ring and In-between-Ring (IBR), are shown below the alignment.

SF1.13. Phylogeny of selected deduced deubiquitinase sequences

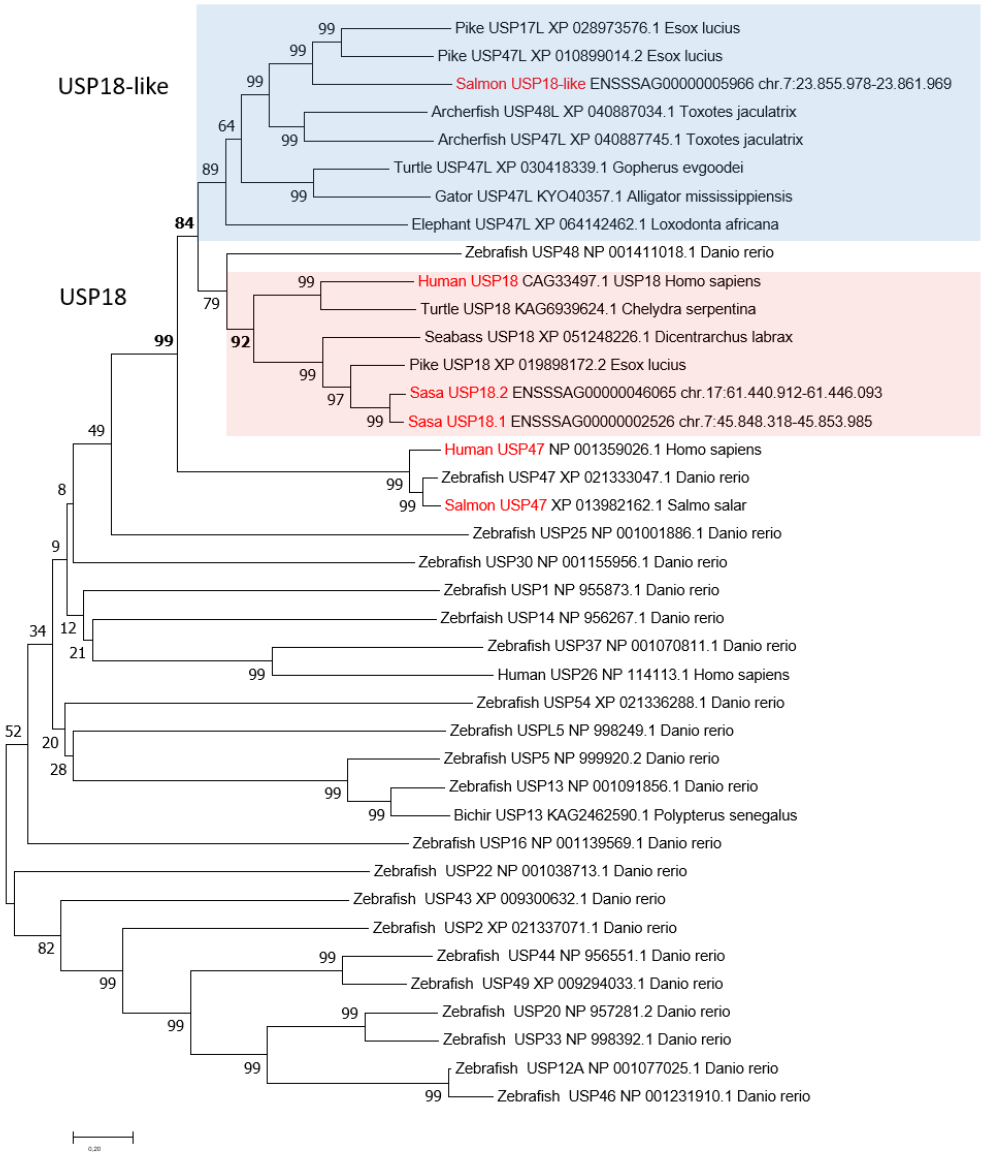

Legend to Figure SF1.13. The evolutionary history was inferred using the Neighbor-Joining method (Saitou N. and Nei M. 1987). The optimal tree with the sum of branch length = 27,24461490 is shown. The percentage of replicate trees in which the associated taxa clustered together in the bootstrap test (1000 replicates) are shown next to the branches (Felsenstein J. 1985). The tree is drawn to scale, with branch lengths in the same units as those of the evolutionary distances used to infer the phylogenetic tree. The evolutionary distances were computed using the Poisson correction method (Zuckerkandl E. and Pauling L. 1965) and are in the units of the number of amino acid substitutions per site. The analysis involved 39 amino acid sequences. All ambiguous positions were removed for each sequence pair. There were a total of 1452 positions in the final dataset. Evolutionary analyses were conducted in MEGA7 (Kumar S., Stecher G., and Tamura K. 2016). Zebrafish sequences from NCBI originate from Tse et al.2009.

SF1.14. Alignment of deduced USP18 amino acid sequences

```
                    *        20         *        40
ssUSP18a : -----------------------------------MARRMFSKCCCSWS :  14
ssUSP18b : ----------------------------------M..G.S..MFIG... :  15
drUSP18  : ----------------------------MGDYWSK..YHRRYIRSS... :  21
hsUSP18  : MSKAFGLLRQICQSILAESSQSPADLEEKKEEDSN.K.EQPRERPRA.D :  49
           *        60         *        80         *       1
ssUSP18a : LPIGSRSEGLRGLMNYGLSCCVNALLQSFSATWELVDLLKGWNPVD--- :  60
ssUSP18b : ...R..LQ.....Q.........................E......--- :  61
drUSP18  : ---SN.GYEV...F.D................T..L.I.NK...S.GIE :  67
hsUSP18  : Y.-----H..V..H.I.QT..L.S.I.V.VMNVDFTRI..RITVPRGAD :  93
           00         *       120         *       140
ssUSP18a : --KESVPLYLRKVLLAMQSDQSQPAPHQDFLRCLDRNYICFHVQQDADE : 107
ssUSP18b : --......................D..K...H.......RN........ : 108
drUSP18  : --QSNI..Q..NT..SVR-.P.HS...KG..D..Y.HA..RYT...... : 113
hsUSP18  : EQRR...FQMLLL.EK..DSRQKAVRPLELAY..QKCNVPLF..H..AQ : 142
             *       160         *       180         *
ssUSP18a : VFLSILNLIQQQMSDKALAQEIHSLYKVTVETYLQCLECTYIETGTSFL : 156
ssUSP18b : ......................Q...N................N..... : 157
drUSP18  : I.HM....SLK..P.HE.....R...TIE...QVT.MD..F.HRLPNSY : 162
hsUSP18  : LY.KLW...KD.IT.VH.VERLQA..TIR.KDS.I.VD.AMESSRN.SM : 191
            200         *       220         *       240
ssUSP18a : LSLPMHISESHD----SLEDCVRFFFKLQELRDGDKCYCEKCGEKKPFK : 201
ssUSP18b : .......R...N----.....I....E.............R......S. : 202
drUSP18  : F...LV.H.GEN----T..S.IKS...K.K.EVSE.FF.DR.EK.Q.CA : 207
hsUSP18  : .T..LSLFDVDSKPLKT...ALHC..QPR..SSKS..F..N..K.TRG. : 240
               *       260         *       280         *
ssUSP18a : QGFKLISLPPVLCLHLKRFRNSHGYTRKLHYKVTFPETLNISEILTAEA : 250
ssUSP18b : .............................CQ.................. : 251
drUSP18  : YEQ..V...QI..V.......E..LIK..DS........K..VFAAGQS : 256
hsUSP18  : .VL..TH..QT.TI..M..SIRNSQ...ICHSLY..QS.DF.Q..PMKR : 289
              300         *       320         *       340
ssUSP18a : LS-ERYVQSDSQYSLCAVIVHSGDAMFGHYTAYVRH-KDQSWYYANDSY : 297
ssUSP18b : ..-.S...............................-............ : 298
drUSP18  : EN-AASDSPQAD...F.....I.S........FI.QTQ..M.......R : 304
hsUSP18  : E.CDAEE..GG..E.F...A.V.M.DS...CV.I.NAV.GK.FCF...N : 338
                 *       360         *       380         *
ssUSP18a : VR------------------------------------QVSWKEVQNTY : 310
ssUSP18b : ..QVRENEWKNVYTTQQVCVMHIPFFCIIKCNITFAQT........... : 347
drUSP18  : .N------------------------------------.AT..D..D.. : 317
hsUSP18  : IC------------------------------------L...EDI.C.. : 351
                400         *       420         *
ssUSP18a : GGSQR--EDTAYMLLYRRTPEGKQQEWSSG------------- : 338
ssUSP18b : ...L.--.E...................T.DGAKGIKSGDWRP : 388
drUSP18  : K.-----RE...L....K----.SLDS-..------------- : 337
hsUSP18  : .NPNYHWQE...L.V.MK------M.C---------------- : 372
```

NCBI sequence references not shown in SF1.13 are USP18 sequences from zebrafish (dr) XP_005164589.1 (first published by Chen et al.(2015)) and human (hs) USP18 NP_059110.2 aligned against Atlantic salmon (ss) sequences.

SF1.15. Differentially expressed Proteasome, MHC and Peptide loading genes

			A	B	C	D	E	
	**Gene**	**Ensembl ID**	**ISAV-4h**	**ISAV-48h**	**C-4h**	**C-58h**	**IFNg-48h**	**Genome location**
MHCI (UDA) chr. 14	TAPBPb	ENSSSAG00000040723	83	202	53	63	158	chr.14:64.331.687-64.338.464
	PSMB8b	ENSSSAG00000041842	187	675	103	94	2067	chr.14:64.459.783-64.462.906
	PSMB12b	ENSSSAG00000041924	31	123	14	12	638	chr.14:64.461.734-64.475.306
	PSMB9b	ENSSSAG00000042126	157	423	113	100	1016	CAJNNT020000527.1:2.714
	TAP2b	ENSSSAG00000077602	388	1414	28	18	1819	CAJNNT020000527.1:6.667
	PSMB10b	ENSSSAG00000049592	204	676	191	203	2085	chr.14:65.322.989-65.329.329
MHCI (UBA) chr. 27	TAPBPa	ENSSSAG00000077407	1244	2826	362	324	15020	chr.27:10.412.120-10.417.481
	UBA	ENSSSAG00000077419	14313	32109	12020	9419	85742	chr.27:10.426.981-10.465.906
	PSMB13a	ENSSSAG00000077561	480	1757	263	239	4869	chr.27:10.521.614-10.527.869
	PSMB12a	ENSSSAG00000077444	1218	3127	894	828	6513	chr.27:10.526.774-10.532.085
	TAP2a	ENSSSAG00000042201	33	152	5	8	537	chr.27:10.536.644-10.543.577
	PSMB10a	ENSSSAG00000078793	173	810	153	191	1170	chr.27:10.968.968-10.979.342
Other regions	PSMA6.2	ENSSSAG00000044595	179	597	121	123	1653	chr.9:122.004.507-122.014.190
	PSMA6.5	ENSSSAG00000052764	348	781	224	202	1421	chr.20:55.247.026-55.250.624
	PSMA7b	ENSSSAG00000045742	33	191	42	28	384	chr.13:23.971.237-23.975.942
	TAP1	ENSSSAG00000005352	862	2506	494	503	5594	chr.5:58.200.693-58.210.551
	TAPBPRb	ENSSSAG00000074475	284	665	284	323	4173	chr.5:72.002.351-72.008.713
	TAPBPL1a	ENSSSAG00000055828	316	1276	150	159	928	chr.9:119.297.563-119.311.982
	TAPBPL2	ENSSSAG00000039745	322	1367	125	144	2179	chr.7: 12.926.074-12.930.490

Average number of reads across all five replicates per group are shown for each gene. Nomenclature is adapted from Grimholt et al. (2018). Genes residing within each of the duplicate the MHCI regions on chromosomes 14 and 27 are shown on the left hand side with genomic location shown on the right hand side.

SF1.16. References:

Chen C., Zhang Y-B, Gui J-F. Expression characterization, genomic structure and function analysis of fish ubiquitin-specific protease 18 (*USP18*) genes, Developmental & Comparative Immunology, 52, 2, 2015. doi.org/10.1016/j.dci.2015.05.003.

Clouthier SC, Rector T, Brown NEC, Anderson ED. Genomic Organization of Infectious Salmon Anaemia Virus. *J Gen Virol* (2002) 83(Pt 2):421-8. doi: 10.1099/0022-1317-83-2-421.

Dannevig BH, Brudeseth BE, Gjoen T, Rode M, Wergeland HI, Evensen O, et al. Characterisation of a Long-Term Cell Line (Shk-1) Developed from the Head Kidney of Atlantic Salmon (*Salmo Salar* L). *Fish Shellfish Immun* (1997) 7(4):213-26. doi: DOI 10.1006/fsim.1996.0076.

Felsenstein J. (1985). Confidence limits on phylogenies: An approach using the bootstrap. Evolution 39:783-791.

Grimholt U. Whole Genome Duplications Have Provided Teleosts with Many Roads to Peptide Loaded Mhc Class I Molecules. *BMC Evol Biol* (2018) 18(1):25. Epub 20180223. doi: 10.1186/s12862-018-1138-9.

Grimholt U, Fosse JH, Sundaram AYM. Selective Stimulation of Duplicated Atlantic Salmon Mhc Pathway Genes by Interferon-Gamma. *Front Immunol* (2020) 11:571650. Epub 20201006. doi: 797 10.3389/fimmu.2020.571650.

Kumar S., Stecher G., and Tamura K. (2016). MEGA7: Molecular Evolutionary Genetics Analysis version 7.0 for bigger datasets.Molecular Biology and Evolution 33:1870-1874

Larkin MA, Blackshields G, Brown NP, Chenna R, McGettigan PA, McWilliam H, Valentin F, Wallace IM, Wilm A, Lopez R, et al. Clustal W and Clustal X version 2.0. Bioinformatics. 2007;23(21):2947–8.

Liu M, Reimschuessel R, Hassel BA. Molecular cloning of the fish interferon stimulated gene, 15 kDa (*ISG15*) orthologue: a ubiquitin-like gene induced by nephrotoxic damage. Gene. 2002 Oct 2;298(2):129-39. doi: 10.1016/s0378-1119(02)00932-0.

Rokenes TP, Larsen R, Robertsen B. Atlantic Salmon Isg15: Expression and Conjugation to Cellular Proteins in Response to Interferon, Double-Stranded Rna and Virus Infections. *Mol Immunol* (2007) 44(5):950-9. Epub 20060511. doi: 10.1016/j.molimm.2006.03.016.

Saitou N. and Nei M. (1987). The neighbor-joining method: A new method for reconstructing phylogenetic trees. Molecular Biology and Evolution 4:406-425.

Tse WK, Eisenhaber B, Ho SH, Ng Q, Eisenhaber F, Jiang YJ. Genome-wide loss-of-function analysis of deubiquitylating enzymes for zebrafish development. BMC Genomics. 2009 Dec 30;10:637. doi: 10.1186/1471-2164-10-637. PMID: 20040115; PMCID: PMC2809080.

Zuckerkandl E. and Pauling L. (1965). Evolutionary divergence and convergence in proteins. Edited in Evolving Genes and Proteins by V. Bryson and H.J. Vogel, pp. 97-166. Academic Press, New York.
